# Serial Chemical Crystallography for Autonomous Quantitative Phase Analysis in an Electron Microscope

**DOI:** 10.1002/smtd.202500889

**Published:** 2025-10-20

**Authors:** Taimin Yang, David Geoffrey Waterman, Zheting Chu, James Beilsten‐Edmands, Zhehao Huang, Xiaodong Zou

**Affiliations:** ^1^ Department of Chemistry Stockholm University Svante Arrhenius väg 16C Stockholm SE‐10691 Sweden; ^2^ Department of Physics and Astronomy University of California Irvine CA 92697 USA; ^3^ STFC, Rutherford Appleton Laboratory Didcot OX11 0FA UK; ^4^ Research Complex at Harwell, Rutherford Appleton Laboratory Didcot OX11 0FA UK; ^5^ Diamond Light Source Ltd. Harwell Science and Innovation Campus Didcot OX11 0DE UK; ^6^ Electron Microscopy Center School of Emergent Soft Matter South China University of Technology Guangzhou 510640 China

**Keywords:** autonomous data collection, beam‐sensitive materials, quantitative phase analysis, serial crystallography, SerialED

## Abstract

We present serial electron diffraction with tilt (t‐SerialED), a method for fast autonomous phase and structural analysis of beam‐sensitive, nano‐sized polycrystalline materials. Unlike traditional workflows collecting datasets crystal by crystal, t‐SerialED acquires datasets using a batch‐by‐batch approach, which speeds up the data acquisition. t‐SerialED combines robust indexing from 3D reciprocal space with still‐shot integration and merging methods from serial crystallography. t‐SerialED enables high‐throughput analysis of beam‐sensitive, multi‐phase mixtures across a wide range of materials, from nanoporous frameworks to pharmaceutical compounds. By resolving key challenges in serial crystallography such as indexing and preferred orientation, this method enables precise structure determination, including the visualization of guest molecules and non‐covalent interactions like hydrogen bonding and proton charge transfer. Demonstrated on a range of samples from nanoporous materials to pharmaceuticals, t‐SerialED expands the capabilities of serial chemical crystallography from single‐phase to complex multi‐phase systems. It can become a complementary method to traditional crystallography methods, offering a robust solution for routine quantitative phase analysis and structure determination.

## Graphical abstract

t‐SerialED, a novel electron diffraction method, enables rapid, high‐throughput structural analysis of nano‐sized materials. By adapting a batch‐by‐batch data collection approach, t‐SerialED integrates 3D reciprocal information to serial crystallography datasets. t‐SerialED combines rotational indexing methods and serial crystallography integration techniques, allowing analysis of complex multi‐phase mixtures and revealing fine structural details such as guest molecules and hydrogen bonding.

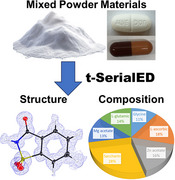

## 1 Introduction

Recent advancements in robotics and artificial intelligence have significantly propelled the development of autonomous synthesis and characterization workflows.^[^ ^1^, ^2^ ^]^ Powder X‐ray diffraction (PXRD) is the dominant method for characterization in these workflows. Nevertheless, challenges arise when a polycrystalline product contains multiple phases, phases with low contents (<5%), phases with similar unit cell parameters and structures with severe peak overlaps in PXRD patterns. As autonomous synthesis can usually produce many samples within a short timeframe, there is a pressing demand for innovative techniques that can match the speed and obtain the compositions and atomic structures for complex mixtures simultaneously.

The last ten years witnessed several emerging technologies to study crystals that traditional X‐ray techniques cannot effectively analyze. X‐ray free‐electron lasers (XFELs) have been crucial in driving the advancement of serial femtosecond crystallography (SFX).^[^ ^3^, ^4^, ^5^ ^]^ This technique captures a single snapshot from each crystal, avoiding dose accumulation associated with rotation series. The method is widely used in macromolecular crystallography. However, when applied to chemical crystallography, the small unit cell leads to sparsity of reflections on each frame, hindering unit cell and orientation matrix determination.^[^ ^6^ ^]^ Although attempts have been made to resolve this problem, the solution generally requires pure samples^[^ ^4^, ^5^, ^6^ ^]^ and is difficult to apply to phase mixtures. Besides, the scarcity and costliness of XFEL beamtime limits this technique as a routine analysis method. Electron microscopes are a comparatively cost‐effective alternative for measuring diffraction patterns from nanosized crystals. Recently, 3D electron diffraction (3DED),^[^ ^7^ ^]^ including stepwise^[^ ^8^, ^9^ ^]^ and continuous rotation geometry,^[^ ^10^, ^11^, ^12^, ^13^ ^]^ has become an reliable method to determine the structure of beam‐sensitive nanosized materials in transmission electron microscopes (TEMs).^[^ ^7^, ^8^, ^10^, ^14^, ^15^, ^16^ ^]^ Several attempts have been implemented to achieve automatic^[^ ^17^, ^18^, ^19^, ^20^ ^]^ or semi‐automatic^[^ ^21^, ^22^ ^]^ structure analysis. However, the data collection speed limits further applications of 3DED in phase analysis due to cost and time.^[^ ^18^, ^22^ ^]^ Therefore, serial electron diffraction (SerialED), a single‐shot‐per‐crystal technique, has been applied to both small‐^[^ ^23^ ^]^ and macro‐molecule crystals.^[^ ^24^ ^]^ Nevertheless, the application of the method is limited by difficulties during data processing: 1) requires a known unit cell; 2) indexing is difficult due to lack of 3D information caused by the flat Ewald sphere of high energy electrons; 3) indexing ambiguity; 4) preferred orientation limits the completeness of the merged data; 5) unable to process an ED pattern from multiple crystals. Consequently, this method remains underutilized and has primarily been applied to crystals with relatively high symmetry and large unit cell parameters.

Here we present SerialED with tilt (t‐SerialED), a multi‐shots‐per‐crystal approach aiming to perform autonomous quantitative analysis for complex mixtures. t‐SerialED collects 3D dataset using a batch‐by‐batch approach, which speeds up the data acquisition process. The full workflow can be conducted in a conventional TEM without customized hardware, making sure the accessibility of the method. This method addresses the challenges in small molecule SFX (smSFX) and SerialED by merging 3D reciprocal space information into the serial crystallography data acquisition and processing workflows. We conducted autonomous data collection and analysis of the compositions and crystal structures across a range of beam‐sensitive and polycrystalline mixtures, spanning from nanoporous materials to pharmaceutical compounds. We demonstrate that t‐SerialED can determine the positions of disordered guest molecules/species in the pore and visualize the interaction between the guest molecule and the framework. We also show that t‐SerialED can accurately determine the positions of hydrogen atoms and study non‐covalent interactions, such as hydrogen bonding and proton charge transfer. By resolving the challenges mentioned above, t‐SerialED extends the analyzing scope of serial chemical crystallography from single phase to complex mixtures. We expect t‐SerialED to become a general method for chemical crystallography and phase analysis of submicron or nano‐sized mixtures, complementing PXRD for routine sample checking and phase analysis.

## 2 Key Concepts

### 2.1 Batch‐by‐Batch Data Collection

**Figure 1:**
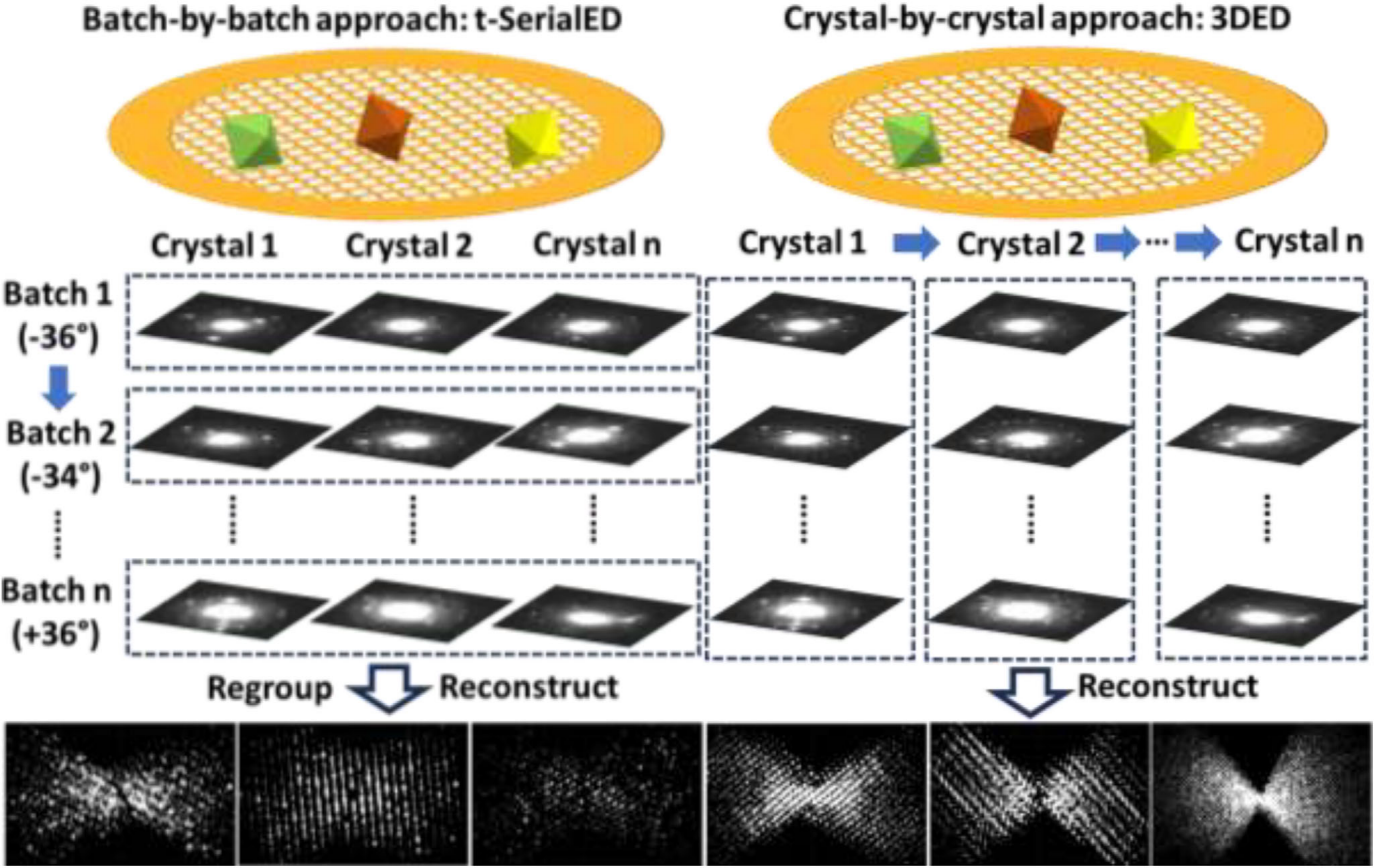
Comparison between the batch‐by‐batch data collection approach with traditional crystal‐by‐crystal approach. The batch‐by‐batch approach collects all targets from one angle, then rotates the stage to the next angle and collects the next batch. The crystal‐by‐crystal approach rotates stage from start to end for one crystal and then move to the next crystal.

Traditionally, 3DED collects tilt series crystal by crystal. A complete tilt series is collected for one crystal before moving to the next crystal. If datasets from ten crystals need to be collected, then the goniometer needs to rotate back and forth ten times, which limits the throughput. To overcome this limitation, we present a new batch‐by‐batch approach, combining the advantages of both 3DED and SerialED. In our method, SerialED is first applied to collect still ED patterns from all crystals in one batch at the same tilt angle. Then the stage is tilted to the next angle and still frames are collected from the same batch of crystals, as shown in **Figure** **1**. After the stage reached a target angle, ED patterns from the same crystal within different batches will be regarded as one tilt series. In this way, the stage only needs to rotate from start to end once for multiple crystals, increasing the data collection efficiency. After collecting all crystals, the collected ED patterns are regrouped into individual stepwise rotation datasets corresponding to each particle ready for unit cell determination and indexing.

### 2.2 Unit Cell Determination from the Stepwise Rotational Datasets

The determination of the unit cell plays a critical role in phase analysis. After regrouping, the batch‐by‐batch approach collects still frames at multiple target angles for each crystal, forming 3D reciprocal lattice for unit cell determination and indexing. Since 3D reciprocal lattice is already available, it is possible to use rotational indexing methods based on Fourier transformation,^[^ ^25^ ^]^ which do not require prior knowledge about symmetry or cell constants. The number of images per crystal can be reduced significantly because only unit cell is required from each rotational dataset. This alleviates the main bottleneck for serial crystallography and allows us to speed up the data collection and processing.

### 2.3 Combining X‐Ray and XFEL Methods to Process Discrete Tilt Series

For individual discrete tilt series with large angle intervals, single crystal X‐ray programs can easily index them. However, these programs are unsuitable for processing these datasets because they assume integrated intensities during data integration and merging. On the other hand, serial crystallography can integrate and merge still‐shot frames, but indexing is challenging, especially for electron diffraction data due to the flat Ewald sphere. Here we combine the strengths from both approaches. After indexing, each tilt series is ungrouped and split into individual frames. The orientation matrix of each frame is calculated by multiplying the orientation matrix of the whole tilt series and the rotatioin matrix calculated from the tilt angle relationship within a tilt series. Then, each frame is treated as an individual crystal and the calculated orientation matrix of each frame is input directly into the serial crystallography programs for data integration and merging.

## 3 Results

### 3.1 Ab Initio Structure Determination of Metal–Organic Frameworks

**Figure 2:**
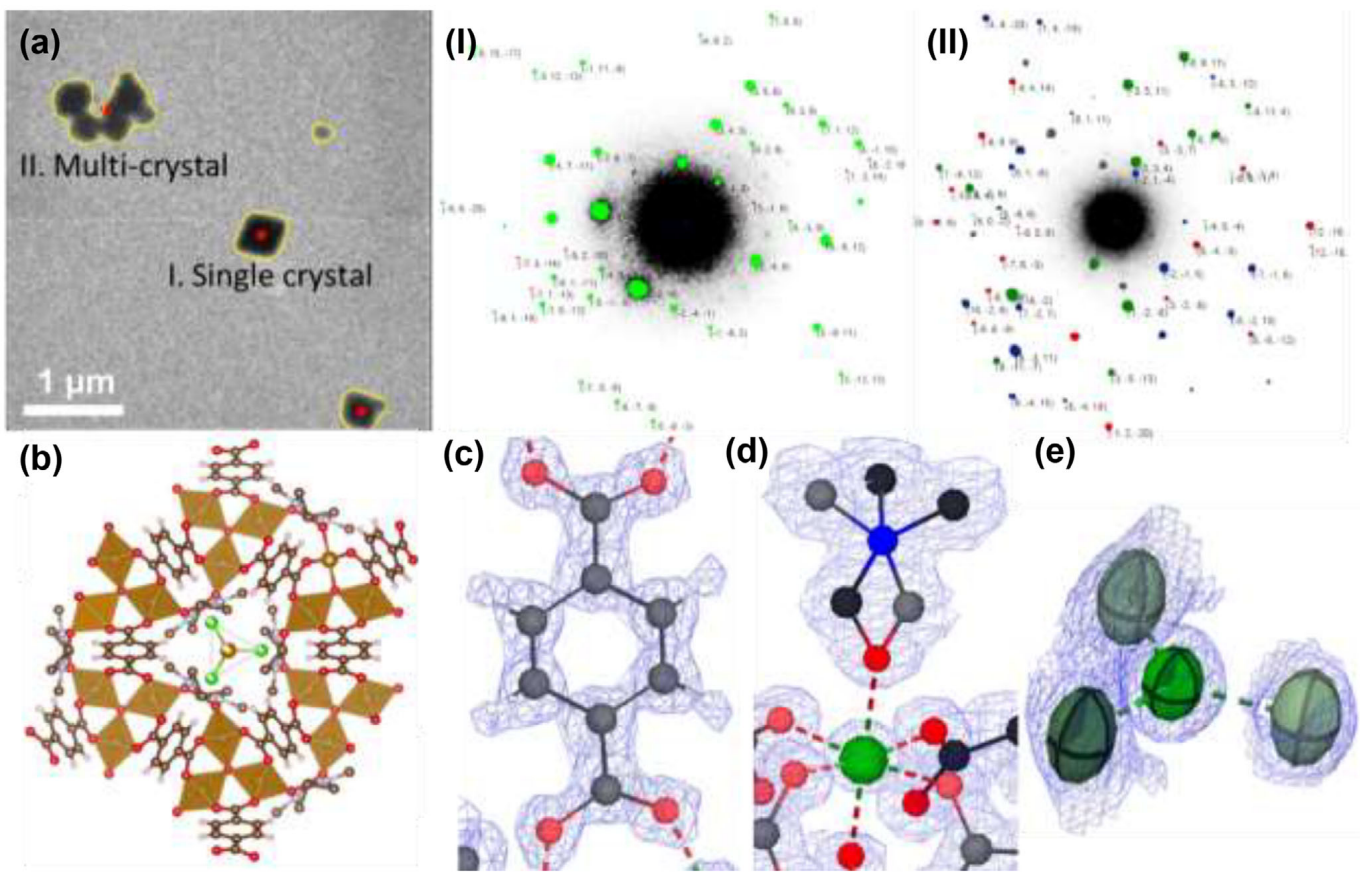
Structure determination of MOF‐235 by t‐SerialED: a) the TEM image shows the crystal morphology and distribution on the grid. The red dots indicate the positions for collecting datasets. Position I is a single crystal and (I) shows the ED pattern from this crystal. The green dots show the indexed spots by a single lattice. Position II reveals the aggregation of multiple nanocrystals and (II) displays the ED pattern from this area. The red, green and blue dots indicate that the ED pattern is indexed by three distinct sets of lattices. b) Refined MOF‐235 structure. Electrostatic potential map of c) hydrogen atoms on the ligand, d) a DMF guest molecule coordinated to a framework Fe atom (the disordered C atoms are shown in dark grey and black C atoms) and e) a FeCl_3_ species in the channel.

We chose MOF‐235^[^ ^26^ ^]^ as an example for structure determination from t‐SerialED datasets. As shown in **Figure** **2**a, some of the crystals are well‐isolated and some of them tend to stick together. The crystal finding algorithm^[^ ^23^ ^]^ will identify both as targets and collect t‐SerialED datasets. The ED pattern from aggregated crystals shows a typical multi‐crystal ED pattern, which is successfully indexed and all three lattices with different orientations are resolved. As aggregated particles are unavoidable for most of TEM samples, inclusion for these areas will not only increase the data collection efficiency but also reduce the bias and systematic error in quantitative phase analysis. We reached a complete dataset with high redundancy by merging 160 datasets collected within ≈ 1 h, containing 159 indexed datasets. We used both *CrystFEL* and *DIALS* to process the datasets and obtained similar results (Table S8, Supporting Information). The 3D reciprocal space was visualized from an individual t‐SerialED dataset and space groups were determined from the systematic absence conditions (Figure S9, Supporting Information). The unit cell distribution analysis is performed after using DIALS to index all the datasets (Figure S10, Supporting Information). The analysis shows very small standard deviations (below 0.5%) for both lengths and angles. As shown in Figure 2b, the crystal structure can be determined ab initio using dual‐space methods in *SHELXT* in the space group of $P\bar{6}2c$ and the structure can be refined anisotropically. The final *R*_1_ reached below 14%, which is comparable to the results from 3DED datasets. The framework atoms are correctly resolved and all hydrogen atoms of the ligand are identified from the difference Fourier map. Moreover, disordered solvent molecules (DMF) attached to the open metal (Fe) site of the framework are clearly visualized. The DMF molecules have two configurations to interact with the Fe site (Figure 2d). The reactant ligands FeCl_3_, captured in the pores, are also identified from the residue peaks (Figure 2e). By comparing with a reference structure determined by single crystal X‐ray diffraction,^[^ ^26^ ^]^ the calculated average deviation from reference atoms (ADRA) and maximum deviation from reference atoms (MDRA)^[^ ^27^ ^]^ is 0.15 and 0.42 Å, respectively, indicating the structure is consistent with the reference structure. The results prove that t‐SerialED is a reliable structure determination method that allows investigation of guest molecules/species and their interactions with the framework.

### 3.2 Phase Analysis and Structure Determination for Complex Mixtures

Phase analysis of polycrystalline materials is crucial for sample screening, synthesis optimization, and quality control. Next, we use a mixture as an example to show the capbility of t‐SerialED in phase analysis. The mixture consists of 6 compounds in low‐symmetry space groups, with a predefined weight percentage (16.7 wt.%). Since the densities of these compounds are different, the volume ratios are calculated (Table S9, Supporting Information). We want to show that t‐SerialED can automatically analyze the phase compositions of the complex mixture and determine the structure at the same time.

**Figure 3:**
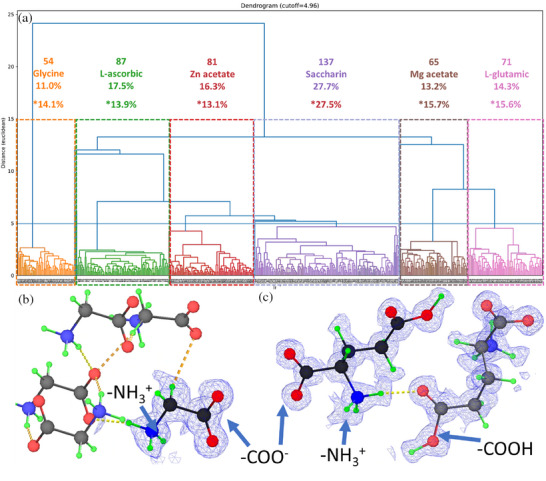
Composition and structure determination results for the complex mixtures: a). Dendrogram showing the compositional analysis results from t‐SerialED and responding structure determined from each unit cell cluster. The *y* axis is the Euclidean distance between the unit cell parameters and is described in (Equation S1, Supporting Information). As indicated by the number of branches (one branch represents one crystal) under each phase, the volume ratio of each compound can be calculated. “*” indicates the expected volume ratio for each compound. The branches that have large deviation to any of the clusters are identified as outliers and excluded in the analysis. Hydrogen bonding network and proton charge transfer in (b). glycine (c). L‐glutamic acid. The green atoms are hydrogen atoms and the yellow dashed lines are hydrogen bonds. The hydrogen atom in carboxyl group (‐COOH) transferred to the amino group (‐NH_2_), forming ‐COO^−^ and ‐NH_3_.

We collected 902 datasets over 6.5 h, with 495 of them successfully indexed (54.9%). The indexing of the rest of the datasets was obstructed by factors such as thick sample, amorphous content, or the polycrystalline nature (Figure S8, Supporting Information). The unit cells were clustered by applying a threshold to the dendrogram, resulting in six distinct clusters (**Figure** **3**a). By counting the number of datasets in each cluster, the relative volume ratio of each compound was calculated. It is also possible to perform phase analysis based on the areas of crystals in the acquired image. However, the image segmentation algorithm still cannot separate aggregates without a clear boundary, which is often the case in real samples. After multiplying with the density of each compound, the weight ratios from t‐SerialED were calculated as follows: 13.3% (glycine), 20.5% (L‐ascorbic acid), 19.7% (zinc acetate), 16.8% (saccharin), 14.3% (magnesium acetate), and 15.4% (L‐glutamic acid). Overall, the phase analysis results show trends consistent with the expected volume ratios, particularly for saccharin, which differed by only 0.2%. The absolute deviations for the other compounds ranged from 1.3% to 3.8%. Several factors could account for these deviations. One possibility is the loss of crystallinity during grinding.^[^ ^22^ ^]^ Another possibility is that some materials may have a higher affinity for the grid support during sample preparation, leading to selective adhesion of crystals. Since we count the number of indexed lattices rather than the actual volume, this could also introduce some errors. Furthermore, the crystal shapes and sizes vary significantly (Figure S13d, Supporting Information), even after crushing. Some crystals are uncrushable and tend to aggregate. For aggregates larger than the beam size, the edges of the aggregates are identified automatically (Figure S4, Supporting Information). The inner parts of the aggregates are ignored because they are very thick and often impenetrable by electrons.

After phase analysis, we integrated and merged frames within each cluster using existing serial crystallography methods, determining the structures ab initio for all compounds. These structures were refined anisotropically, with hydrogen atoms in four compounds identified from difference Fourier maps (Figure 3; Figure S11, Supporting Information). The clear identification of hydrogen atoms allows us to investigate structural details in glycine and L‐glutamic acid. Notably, Figure 3b,c shows the hydrogen bonding and proton transfer in these two amino acids. While no residue peak can be found around the carboxyl group, three residue peaks are found near the amino group arranged in tetrahedral geometry, indicating the proton transfer from ‐COOH to ‐NH_2_ group. In saccharin, the hydrogen atom is positioned next to the nitrogen atom in the five‐membered ring, confirming the dominant tautomeric form after crystallization.^[^ ^28^ ^]^ All datasets achieve relatively high completeness. Most of the samples reached a completeness of nearly 100% and the dataset of Zn acetate has a completeness of 88.6%, which is the lowest among the six compounds (Table S11, Supporting Information). The *CC_1/2_* plot (Figure S12e, Supporting Information) for Mg acetate shows low correlation, and the observed resolution of ED patterns for Mg acetate is lower. Consequently, the number of merged frames for Mg acetate is around 330, which is the lowest among the six compounds. The reduced frames lead to partial reflections and poor intensity estimations, resulting in the highest *R*_1_ value (33.3%) among the six compounds. For the other compounds, *R*_1_ values range from 16.1% to 22.6%, which fall within the normal range for 3DED datasets.

### 3.3 Phase Analysis for Pharmaceutical Tablets and Capsules

We applied t‐SerialED to a tablet and a capsule (Figure S13, Supporting Information), in formulations of paracetamol and ferrous glycine complex (**FGC**), respectively. The drug formulations contain a variety of non‐active agents such as binders, disintegrants and wax. According to the registration information (Table S3, Supporting Information), both drugs contain a tiny amount of highly crystalline talc (Mg_3_Si_4_O_10_(OH)_2_), but the weight ratio is unknown. Other non‐active contents, such as starch and hypromellose, are unlikely to diffract to enough resolution.

### 3.4 Paracetamol (One Polymorph)

**Figure 4:**
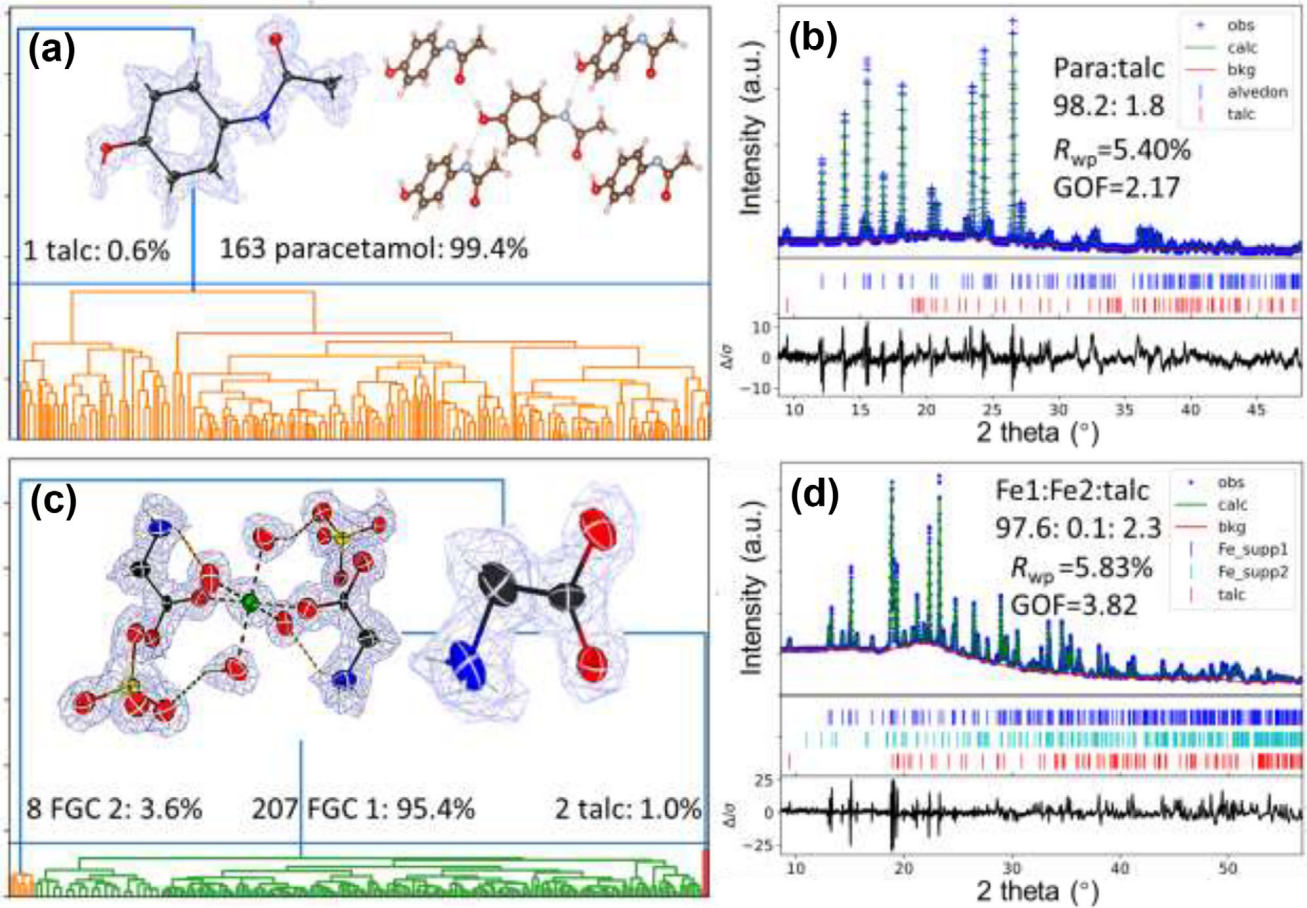
Comparison of phase analysis results from t‐SerialED and PXRD Rietveld refinement for a,b) paracetamol tablet and c,d) iron supplement capsule. The results from t‐SerialED show the relative volume ratio of paracetamol and talc is 99.4:0.6, while the volume ratio of FGC form 1, form 2, and talc is 95.4:3.6:1.0.

We collected 197 datasets in 1.7 h, with 163 of them indexed to the unit cells of the active pharmaceutical ingredient (API), indicating a volume ratio of 82.8% (Table S3, Supporting Information). This deviates from the expected API volume ratio by just 1.4% (81.4%), demonstrating high accuracy considering the various influencing factors discussed above. Additionally, one dataset was indexed to the unit cell of talc, representing 0.6% of the crystalline contents in the tablet. Compared with the analysis from PXRD (**Figure** **4**b), the result differs by 1.2% (PXRD: 1.8%). After phase analysis, we treated every frame from API as separate crystals and applied the serial crystallography approach to integrate and merge these datasets. From the HKL files, we solved the structure ab initio and refined the structure anisotropically with final *R*_1_ of 17.2% (Table S14, Supporting Information). We also located all the H atoms in the structure and visualized the hydrogen bonding network (Figure S14c, Supporting Information).

### 3.5 Ferrous Glycine Complex (FGC, Two Polymorphs)

For **FGC**, 497 datasets were collected within 3.8 h. Among 497 datasets, 215 datasets were indexed to the API unit cells. The volume ratio of APIs in the pellet was determined to be 43.4%, which is 6.2% less than the expected ratio (49.6%). One possible explanation for this discrepancy is the low crystallinity of the API. As shown in Figure S17 (Supporting Information), more than 50% of the datasets have fewer than 150 reflections, whereas for the paracetamol datasets, only 10% of the datasets have fewer than 150 reflections. Further evidence of the API's low crystallinity is the significantly longer time required for PXRD data collection. It took 28 h for the FGC sample, compared to 5 h for the paracetamol sample, to achieve similar counting. Also, the lump near 22° in PXRD pattern of **FGC** indicates a large amount of amorphous content while in the PXRD pattern of paracetamol, the amorphous peak is not so pronounced. From the clustering result (Figure 4c), we conclude that the FGC has two polymorphs, denoted as **FGC** form 1 and **FGC** form 2. They correspond to two polymorphs of **FGC** in CCDC: GLYCFE01 (form 1) and UDOPIO01 (form 2). Of the 215 datasets, 207 (95.4%) were indexed to the unit cell of form 1, while 8 (3.6%) were indexed to the unit cell of form 2. We use PXRD as a reference, since the ratio of different polymorphs is not provided in the drug registration files. Please bear in mind that due to the difference of crystallinity, the result from PXRD still may exhibit significant deviation with the real situation. In this sample, the strong peaks of **FGC** form 2 are submerged in the background because of the low crystallinity (Figure S18, Supporting Information). Consequently, the content of FGC form 2 estimated from PXRD is very low (0.1%), which may not be reliable. For the highly crystalline content, talc, the strongest PXRD peak (9.7°) is clearly visible, indicating that talc constitutes 2.3% of the crystalline content. In contrast, t‐SerialED identified only 2 datasets with the talc unit cell out of 217 indexed datasets (1%). Since both methods can exhibit significant variations when measuring trace amounts of minor phases, it is difficult to determine which result is more accurate. Nonetheless, we conclude that t‐SerialED provides consistent results with PXRD for highly crystalline phases and can also detect minor phases with trace amount, which is often difficult for PXRD due to insufficient number of peaks. Given the shorter acquisition time (3.8 h vs 28 h) and the automated nature of t‐SerialED, we expect this method to complement PXRD by enabling comprehensive analysis of all sample components, regardless of the crystallinity.

Finally, we determined the structure of **FGC** form 1 ab initio and assessed the impact of the number of datasets on data quality. By filtering datasets with fewer than 200 and 100 indexed reflections, we integrated and merged 39 and 93 datasets, respectively. The data processing statistics for both groups are similar, but the final *R*_1_ value decreases by 3.78%, from 20.82% to 17.04%. This result demonstrates that including more datasets with sufficient indexing can significantly improve the structure due to increased data redundancy.

## 4 Discussion

### 4.1 Comparison with SFX

Due to the sparsity of reflections, SFX for small molecules identifies unit cells by aggregating spot‐finding results into high‐resolution powder diffractograms and generating candidate unit cells from the synthetic powder pattern.^[^ ^6^ ^]^ However, this method struggles to provide accurate unit cell information when the sample contains a mixture of several phases. In contrast, t‐SerialED can quantitatively analyze complex mixtures and accurately determine the structure of each phase. Moreover, images are acquired during t‐SerialED data collection, providing valuable insights into particle size distribution, surface features, and internal morphology. t‐SerialED can be adaptable to various types of sample holders, such as the cryo‐holders used in this study, and can be easily applied to other in situ TEM holders, enabling the study of structural dynamics under different environmental conditions.

### 4.2 Comparison with SerialED

The bottleneck of SerialED is indexing. Due to the short de Broglie wavelength of electrons (0.0197 Å at 300 kV), the Ewald sphere is almost flat. Therefore, currently all indexing algorithms, such as TakeTwo,^[^ ^29^ ^]^ FELIX,^[^ ^30^ ^]^ problematic,^[^ ^23^ ^]^ SPIND,^[^ ^31^ ^]^ or PinkIndexer,^[^ ^32^ ^]^ require predefined unit‐cell parameters as restraints since the information along the incident beam direction is missing. Another challenge arises when an ED pattern contains reflections from multiple crystals, as current indexing algorithms are unlikely to provide a correct solution. The incorporation of 3D reciprocal information in *t‐SerialED* datasets enables the use of traditional X‐ray crystallography programs, improving the indexing rate to nearly 100% even for multi‐crystal patterns. This enhances both the number of processable datasets obtained during a TEM session and the accuracy of phase analysis. By collecting ED patterns at various angles, sample preferred orientation can be mitigated, allowing for the acquisition of a complete dataset from fewer crystals. Notably, most of our samples have much lower symmetry compared with current SerialED examples. t‐SerialED can obtain complete datasets from a small number of crystals. Additionally, t‐SerialED does not require prior knowledge about unit cells, enabling phase analysis and structure determination for unknown phases.

Similar to SerialED, the data quality of t‐SerialED drops when the number of merged frames decreases. For example, the final *R*_1_ for the Mg acetate dataset increases to 33% when the number of merged frames dropped to 330 and the fine details in the structure, such as hydrogen atoms, cannot be resolved from the dataset. In addition, the ADP ellipsoids become extremely large and elongated. Even though the completeness is relatively high, the missing fine details in the structure indicate the influence of redundancy. Low redundancy led to low precision in intensity estimation. The issue can be compensated by merging more crystals, as shown in the case of MOF‐235, saccharin and paracetamol.

### 4.3 Comparison with 3DED

3DED has become an effective method for structure determination for beam‐sensitive materials.^[^ ^28^, ^33^, ^34^, ^35^ ^]^ Since fully‐integrated Bragg spots can achieve far better intensity estimation than partial reflections, methods with continuous rotation or precession (PEDT, FAST‐ADT, cRED, cPEDT, etc)^[^ ^10^, ^12^, ^13^, ^36^, ^37^ ^]^ quickly dominated over still‐shot methods (EDT, ADT, RED, etc).^[^ ^8^, ^9^ ^]^ However, for still‐shot methods, even though the intensity estimation of partial reflections is poor, the unit cell can still achieve an accuracy comparable to that from a continuous tilt series. 3DED with continuous rotation/precession collects fully‐integrated Bragg spots to achieve high quality intensity while t‐SerialED uses discrete tilt still series and collects less frames over similar tilt range to increase the throughput. Additionally, for t‐SerialED, the time required for preparation stage is reduced and the manual crystal picking step is eliminated, further increasing the acquisitionthroughput. In addition, due to the high‐throughput nature, t‐SerialED can process ED patterns generated from multi‐crystals, which are considered low‐quality for 3DED datasets and often filtered during data processing. t‐SerialED datasets can also be collected from the edges of large crystals, ensuring optimal data quality even for thick crystals. In previous semi‐^[^ ^22^ ^]^ or fully‐^[^ ^17^ ^]^ automatic 3DED experiments, however, these large and thick crystals are often filtered.

It is commonly believed that continuous rotation geometry can achieve far better structure determination than discrete tilt still series in terms of both *R*_1_ and final structure. Our results show that discrete tilt series can achieve comparable results to 3DED. Structural details, such as the hydrogen bonding network and proton charge transfer, can be revealed by the discrete tilt series. The R‐factors for most of the samples fall within the range between 14% to 25%, which is the normal *R*_1_ range of 3DED. Even though data quality indicators, such as *R_merge_*, *R_meas_*, *CC_1/2_* indicate a poor quality of t‐SerialED datasets, the final *R*_1_ and the structures are comparable to 3DED. For instance, the *R_merge_* for MOF‐235 (Table S7, Supporting Information) and paracetamol (Table S13, Supporting Information) reached 106.1% and 97.8%, respectively. However, the final R‐factor for MOF‐235 and paracetamol reached 14% and 17% and the final structures are reasonable. For MOF‐235, solvent molecules DMF and inorganic species FeCl_3_ are found inside the nanochannel. The hydrogen atoms can even be identified from the residue peaks for both samples. Another example is the *R_merge_* and *CC_1/2_* value of FGC sample is 77.5% and only 75.2%, respectively, which both indicate very bad data quality. Surprisingly, the final *R*_1_ is around 17%, which falls within the normal final *R*_1_ range of 3DED datasets. We also noted that other data quality indicators, such as *R_pim_* and *R_split_*, stay within the normal range for electron diffraction. As these two indicators take the redundancy into account,^[^ ^38^, ^39^ ^]^ the data quality can be better evaluated with these indicators because of the high‐redundancy of t‐SerialED dataset.

Another important aspect is compensation for dynamical scattering. Some of our datasets are collected from large, thick crystals, and the structure refinement using these datasets encountered some difficulties. Even though our crystal finding algorithm identifies the edges of these crystals and collects datasets from the edges, which are relatively thin areas of these crystals, dynamical scattering still negatively affects the structure. Unfortunately, current dynamical refinement programs are unable to handle still frames because they require integrated intensities. Incorporation of precession may help to solve this issue. Since precession can provide integrated intensity, the intensity estimation could also be improved. However, this is beyond the scope of this paper and will be investigated in the future.

## 5 Conclusion

In summary, by incorporating 3D information in SerialED, t‐SerialED resolves the major challenges faced by serial chemical crystallography. The t‐SerialED approach established in this study expands serial electron diffraction as a quantitative analytical method well beyond a structural determination approach. The data collection process requires minimum human intervention after initial setup, making it suitable to run during less busy microscope shifts. With the ability to collect and analyze vast amounts of data, t‐SerialED can be used for compositional analysis, providing a fast, reliable and statistically significant quantitative analysis. We expect t‐SerialED to have a significant impact on the exploration of a wide range of materials, including minerals, zeolites, ceramics, MOFs and pharmaceutical compounds.

## 6 Experimental Section

### Data Acquisition

**Figure 5:**
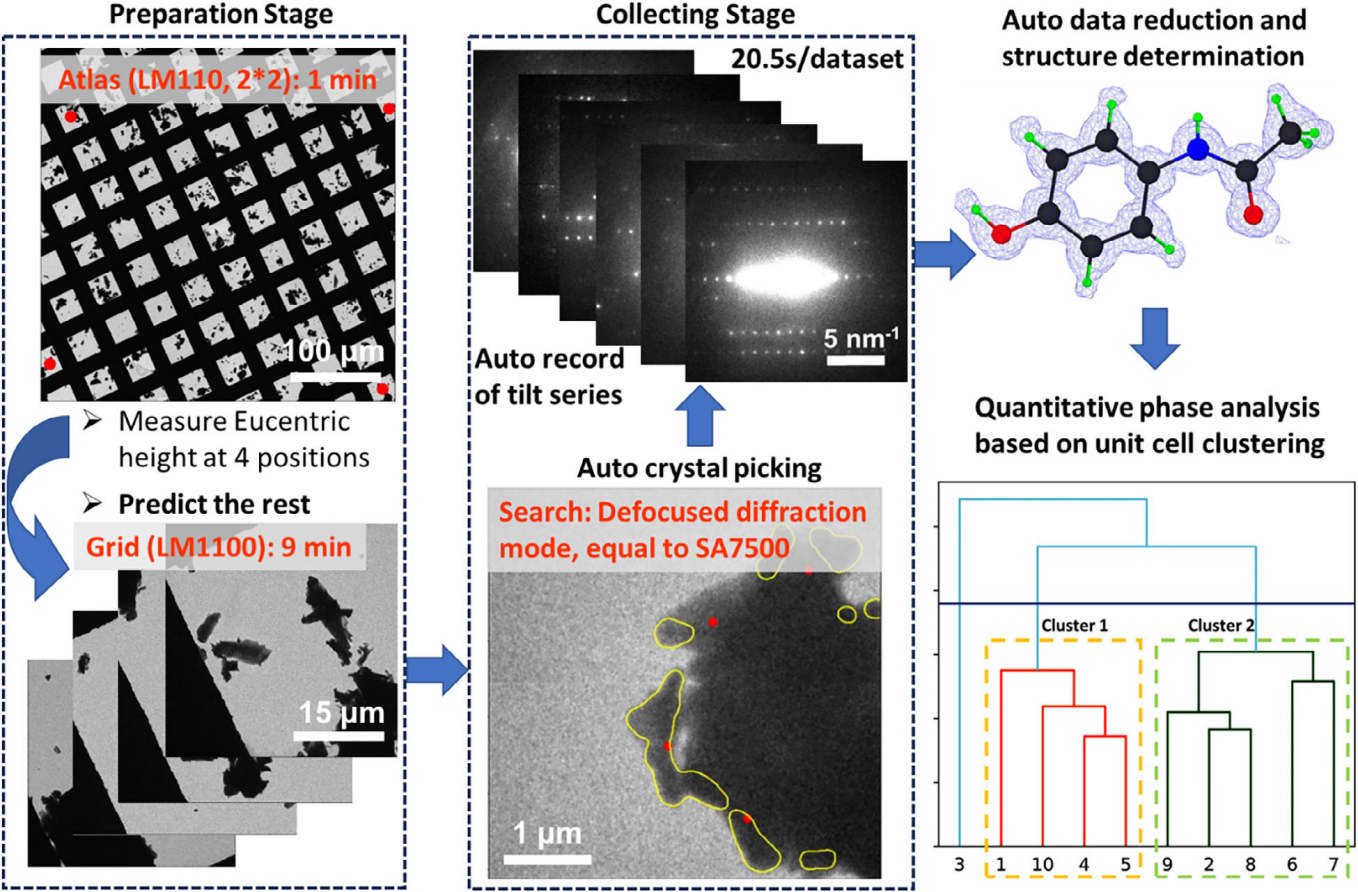
Schematics to show the autonomous workflow of t‐SerialED for structure determination and quantitative phase analysis.

The t‐SerialED experiment is implemented in the software platform *Instamatic* developed by the group.^[^ ^40^ ^]^ **Figure** **5** is a schematic diagram to show the workflow. The experiment can be divided into two stages, preparation stage and collection stage. In the preparation stage, the microscope runs in LM mode to identify the grid holes and measure the eucentric z‐height automatically using an established algorithm in cryo‐tomography.^[^ ^41^ ^]^ The duration depends on how large the low‐magnification montage is collected and the number of points measured for eucentric height. We summarized our setup for the preparation stage and the corresponding time required in Table S1 (Supporting Information). During the collection stage, the size of C2 aperture is set to 50 µm and the microscope keeps running in diffraction mode, avoiding switching the microscope back‐and‐forth from imaging and diffraction modes. A Ronchigram is shown by adjusting the diffraction defocus to identify crystals using a threshold based algorithm.^[^ ^23^ ^]^ Then ED patterns are collected using the batch‐by‐batch approach.

### Data Processing—Data Pre‐Processing

The raw datasets are saved by *Instamatic* ^[^ ^40^ ^]^ in *MRC* format with *REDp* ^[^ ^9^ ^]^ software input file (.*ed3d* format). *REDp* software can be used for 3D reciprocal space visualization. During data acquisition, the center of diffraction pattern will move several pixels as the electron beam tracks the crystal. The center drift will be corrected by cross correlation. After the center drift correction, the *MRC* files are converted to *SMV* files for *DIALS* data pr or *HDF5* files for *CrystFEL* data processing.

### Indexing and Integration

Bragg reflections are identified using the extended dispersion threshold algorithm in *DIALS*.^[^ ^42^ ^]^ Then the unit cell in 3D reciprocal space is calculated using the 3D FFT algorithm in *DIALS*.^[^ ^43^ ^]^ The orientation matrices for all frames in a tilt series are calculated by multiplying the initial orientation matrix at 0° by the rotation matrix, derived from the tilt angle and rotation axis direction.

After obtaining the orientation matrix for each frame, the integrated image intensity at each predicted Bragg peak position is determined using either a background‐subtracted summation algorithm in *CrystFEL* or an ellipsoid partiality modelling plus background‐subtracted summation in *dials.ssx_integrate*.^[^ ^44^ ^]^ For still‐shot data collected with a monochromatic beam, reflections are only partially observed, determined by the intersection of the Ewald sphere and the intensity distribution of a reflection in reciprocal space. This means the reflections measured on an image must be corrected by partiality factors, which depend on the shape of the reflection and the offset between the Ewald sphere and reflection center. *dials.ssx_integrate* ^[^ ^44^ ^]^ refines the crystal model and an ellipsoidal mosaicity model for the spot shapes in reciprocal space that best describes the observed pixel intensity distributions in the strong diffraction spots while simultaneously modelling the Ewald sphere offsets. MOF‐235 datasets are processed by both *CrystFEL* and *DIALS* while the rest of the samples are processed by *DIALS*.

### Merging and Structure Determination

We use existing programs for serial crystallography, such as *partialator* ^[^ ^45^ ^]^ *or xia2.ssx_reduce*,^[^ ^44^ ^]^ to merge the datasets, yielding a plain‐text Shelx HKL file containing the fully reduced reflections. Integrated datasets are scaled and merged by *xia2.ssx_reduce*,^[^ ^44^ ^]^ including indexing ambiguity resolution, without further adjusting partiality estimates. The partiality model used by the SSX tools in *DIALS* is based on an ellipsoid model for the reflections in reciprocal space for each pattern. The parameters of this partiality model and crystal model are refined against the observed pixel data, accounting for Ewald sphere offsets. This is a more sophisticated approach than traditional SFX methods and has been shown to result in improved crystal models, reflection predictions and partiality estimates. If the sample is a pure phase, all datasets can be merged directly. For phase mixtures, the unit cell is first clustered by Eucledian distance and then the datasets in each cluster are merged into an individual HKL file. Ab initio structure solution is conducted using *ShelxT*.^[^ ^46^ ^]^ Structure refinement and visualization of the structure models are performed using *ShelxL*,^[^ ^47^ ^]^ *ShelXle*,^[^ ^48^ ^]^ and *VESTA*.^[^ ^49^ ^]^

## Conflict of Interest

The authors declare no conflict of interest.

## Author Contributions

T.Y. performed conceptualization and planning for the whole project, investigation, implementation, programming, sample synthesis, data acquisition, data analysis, manuscript writing and revision, and funding acquisition. D.G.W. performed data analysis, programming, and manuscript revision. Z.C. performed sample synthesis and data analysis. J.B.‐E. performed programming and manuscript revision. Z.H. performed funding acquisition and manuscript revision. X.Z. performed funding acquisition and manuscript revision.

## Supporting information



Supporting Information

Video S1

## Data Availability

The data that support the findings of this study are openly available in Zenodo at https://doi.org/10.5281/zenodo.13924322, reference number 13924322.
