# Supplementary material for: Serial Chemical Crystallography for Autonomous Quantitative Phase Analysis in an Electron Microscope
Source: Small Methods. 2025 Oct 20;9(12):e00889. doi: 10.1002/smtd.202500889 (PMC12716211; doi:10.1002/smtd.202500889)
Supplement: Supplementary file 1 — Supporting Information [file SMTD-9-e00889-s001.docx]

Serial chemical crystallography for autonomous quantitative phase analysis in an electron microscope

**Taimin Yang^a,b,*^, David Geoffrey Waterman^c,d^, Zheting Chu^a^,  James Beilsten-Edmands^e^, Zhehao Huang^a,f^, Xiaodong Zou^a^**

a Department of Chemistry, Stockholm University, Svante Arrhenius väg 16C, Stockholm, SE-10691, Sweden

b Department of Physics and Astronomy, University of California, Irvine, California 92697, United States

c STFC, Rutherford Appleton Laboratory, Didcot OX11 0FA, UK

d Research Complex at Harwell, Rutherford Appleton Laboratory, Didcot OX11 0FA, UK

e Diamond Light Source Ltd, Harwell Science and Innovation Campus, Didcot OX11 0DE, UK

f Electron Microscopy Center, School of Emergent Soft Matter, South China University of Technology, 510640 Guangzhou, China

* Corresponding email: [taimin.yang@mmk.su.se](mailto:taimin.yang@mmk.su.se)

1. Supplementary Methods

1.1 Sample preparation

MOF synthesis: Terephthalic acid (98%, Thermo Scientific), iron (III) chloride hexahydrate (98%, Thermo Scientific), and dimethylformamide (DMF, 99.8%, VWR chemical). The MOF sample was prepared following a modified method of a previously described procedure^1^. Terephthalic acid (3 mmol) was dissolved in 15 mL of DMF. Thereafter, an equivalent amount of iron (III) chloride hexahydrate (3 mmol) was dissolved in the terephthalic acid/DMF solution. The solution was then transferred to a reactor (reactor volume: 15 mL) that was equipped with an ultrasonic generator probe (VCX150, Sonics & Materials, Inc.). The device was set to continuous mode. This experiment was performed at 60% of the maximum power for 2h.

Complex Mixture: The compounds were carefully weighed by an analytical balance (VWR) and then crushed into fine powder by a mortar. The mixture was prepared as by mixing the following 6 compounds: glycine (99%, Sigma Aldrich), L-ascorbic acid (99%, VWR chemical), Zn acetate (98%, Sigma Aldrich), saccharin (99%, Merck), Mg acetate (98%, VWR chemical), L-glutamic acid (99%, Sigma Aldrich). The input weight is set to 50mg for each compound. By considering the density, the expected volume ratios become: 14.1%, 13.9%, 27.5%, 15.7%, 15.6%.

Pharmaceuticals: Paracetamol tablets (Alvedon, API: 500mg, total: 590mg), Fe supplements capsule (Niferex, API: 567mg, total: 703mg) were purchased from a local pharmacy. The total weight of the medicine was measured by an analytical balance. The weight of API is documented in the manual while the weight of non-API is unknown. We use PXRD as a golden standard to measure the ratio of diffracting non-API content. The API and non-API contents are obtained from the Swedish Medical Products Agency, as shown in Table S3.

1.2 Grid Preparation

The copper grids with continuous carbon support (300-mesh, ultra-thin carbon layer, EMS Inc.) are pretreated with glow-discharge plasma at 15 mA in negative mode using a PELCO easiGlow (Ted Pella Inc.). The glow discharge time is 60 seconds for all samples. For the MOF-235, the sample is dispersed in ethanol and then one drop of the suspension is dipped onto the grid. After drying, the grids are loaded onto a TEM holder. For other samples, the powder is grinded in a mortar for a few seconds and then 1 mg of powder is transferred to a 10 mL glass test tube and mixed with the grid. After shaking the tube, the grids are taken out and loaded onto a TEM holder, where the particles adhere to the carbon film through electrostatic forces.

1.3 Instruments

**TEM:** All the datasets were obtained on a Thermo Fisher Scientific (TFS) Themis-Z electron microscope operated at 300kV with a 4096 × 4096 pixels Gatan Oneview CMOS detector (15 × 15μm pixel size) and a 512 × 512 pixels ASI Medipix 3 hybrid pixel detector (55 × 55 μm pixel size). The microscope was operated in diffraction mode during the whole data acquisition process. A monochromator was used to adjust the electrons drawn from the Schottky type electron gun (X-FEG). The exposure time was set to 0.25s and 0.5s for each imaging frame and ED frame, respectively. During t-SerialED data collection, the tilt range of each crystal was from -36° to +36°. Within the tilt range, the number of still frames taken for each crystal at different angles was set to 7, 25 or 49 and the angle interval of neighboring frames was 12.0°, 3.0° and 1.5°, respectively. When collecting ED frames, the beam size was set to 900 nm and the total current of the beam is 2pA, leading to 0.15 e / Å^2^ s^-1^ in dose rate. When collecting searching images, the beam diameter was set to 8000 nm and the total current is 5pA, resulting in a dose rate of 0.005 e / Å^2^ s^-1^. The accumulated dose depends on the angle interval, as listed in Table S4. Using smaller tilt interval (1.5°) can obtain more frames (49 frames) and reflections, increasing the precision in unit cell determination and the chance to successfully index a multicrystal dataset. However, collecting more frames requires higher dose and longer acquisition time. Therefore, in all the experiments, we chose the medium-speed setup (3°, 25 frames per crystal) to achieve balance between indexing accuracy and acquisition time. The total acquisition time per crystal was 20.5s including the overhead time, which contains crystal finding, adjusting eucentric height, switching modes, moving the beam, rotating the stage and translating the stage. As shown in Table S5, compared with other existing methods, our approach reduces the overheads and makes sure more than 60% of the time and more than 99% of the total dose is spent on collecting ED patterns, making the method timesaving and dose-efficient. The total dose for the medium-speed setup is around 1.9 e / Å^2^, which is even lower than the optimal dose (2.6 e / Å^2^) of lysozyme^2^. In addition, we checked the tilt series and did not find appreciable resolution decrease (Figure S9e-h). Therefore, the dose should be suitable for our sample. The microscope’s beam deflectors are synchronized with the camera through program control. t-SerialED experiments are performed using Instamatic.

For MOF-235 samples, the t-SerialED experiments were conducted under room temperature conditions using a Fischione model 2020 tomography holder. For the other samples, the t-SerialED experiments were conducted under cryogenic temperature (95K) using a high-tilt cryo-transfer holder (Gatan, Model 932).

Our *t-SerialED* approach uses a batch-by-batch approach to collect datasets, similar to SerialED. However, we expose each crystal at multiple rotation angles and obtain the 3D reciprocal lattice for each crystal. The 3D reciprocal lattice is used for indexing unit cell using the indexing algorithms available in 3DED. Then the indexed still frames are merged using existing algorithms in XFEL. Our *t-SerialED* approach can autonomously work on crystals with large size distribution randomly (Figure S1a-d).

Figure S1a shows the preparation stage. A low-magnification overview montage of the grid is captured, and the central positions of the grid squares are identified through automatic image analysis. Then after measuring eucentric heights at selected squares at medium magnification using an algorithm developed in cryo-tomography, the eucentric heights of other places over the montage are predicted using an interpolation algorithm. Even when the grid is bent (Figure S 2), the predicted eucentric height surface can show the curvature of the grid and compensate for the bending, ensuring the robustness of the method. Figure 1b and 1c present the collection stage. An image that shows a position is recorded at high magnification with ~1% of the total dose, and the positions of the crystals are automatically identified (Figure S 3). The algorithm detects isolated crystals, aggregated crystals, large bulks and tracks the edges of the larger crystals, as illustrated in Figure S 4. Still ED patterns are recorded for all identified targets in the image. After all the ED patterns are collected at this angle, collection of one batch is completed. Then the stage tilt to the next angle and the collection for the next batch starts. This workflow achieves a nearly 100% hit rate (crystals successfully hit by the electron beam) and a data collection rate of up to 180 datasets per hour (medium-speed setup, 25 frames). When the data collection for one patch is finished, the stage is moved to a new area, repeating the above process until sufficient datasets are gathered. The 3D reciprocal space (Figure 1d) for each crystal is visualized individually, and the unit cell and orientation matrix are calculated for each frame. Finally, the reflections on each frame are integrated and merged using existing algorithms in XFEL.

**PXRD:** PXRD patterns were recorded using a Bruker D8 Discover X-ray diffractometer (Bruker, UK), using Cu Kα radiation (λ = 1.54184 Å) in 40 kV and 40 mA conditions. Glass capillaries were used to load the sample. The diffractometer collected data from 5° to 60° (2θ) with a scanning speed of 0.2°/min (paracetamol) and 0.033°/min (Fe supplement) at room temperature using Debye-Scherrer geometry. The total data collection time was 5h (paracetamol) and 28h (Fe supplement). Rietveld refinement was performed to analyze the crystalline contents in the paracetamol tablets and iron supplement capsule.

1.4 Clustering of unit cell parameters

Unit cell parameters are clustered by calculating the Euclidean distance, denoted as $d\left( i,j \right)$, between unit cells $i$ and $j$. Datasets are grouped based on this distance metric, with each cluster comprising datasets that have similar unit cell parameters and are considered to represent the same phase.

| $d\left( i,j \right)=\sqrt{\Delta a^{2}\left( i, j \right)+\Delta b^{2}\left( i, j \right)+\Delta c^{2}\left( i, j \right)+k*\left[ \Delta\alpha^{2}\left( i, j \right)+\Delta\beta^{2}\left( i, j \right)+\Delta\gamma^{2}\left( i, j \right) \right]}$ | Equation 1 |
| --- | --- |

In Equation 1, $k$ is a scaling parameter that is defined by the user to adjust the weighting between unit cell length and angle. By default, the value is set to 1. $a_{i}$, $b_{i}$, $c_{i}$, $\alpha_{i}$, $\beta_{i}$ and $\gamma_{i}$ correspond to the unit cell parameters from the ith dataset, where *a*, *b*, *c* are the unit cell lengths and *α*, *β*, *γ* are the unit cell angles. To resolve the angle ambiguities, angles larger than 90° will be transformed to the corresponding angle less than 90°. $a_{j}$, $b_{j}$, $c_{j}$, $\alpha_{j}$, $\beta_{j}$ and $\gamma_{j}$ are the parameters from the jth dataset. $\Delta a^{2}\left( i, j \right)$ is defined as ($a_{i}$ - $a_{j}$)^2^. The same applies to $\Delta b^{2}\left( i, j \right)$ and $\Delta c^{2}\left( i, j \right)$.

The following are methods for calculating the distance between the newly formed cluster $u$ and each $v$. $v$ is the remaining cluster in the forest that is not $u$. The first one is called “average”. It uses the following formula to calculate distance:

| $d\left( u,v \right)=\sum_{ij} \frac{d\left( u\left[ i \right], v\left[ j \right] \right)}{\left( \left\vert u \right\vert*\left\vert v \right\vert\right)}$ | Equation 2 |
| --- | --- |

For all points $i$ and $j$ where $\left| u \right|$and $\left| v \right|$ are the cardinalities of clusters $u$ and $v$, respectively.

The second method uses the Ward variance minimization algorithm. The new entry $d\left( i,j \right)$ is computed as follows,

| $d\left( u,v \right)=\sqrt{\frac{\left\vert v \right\vert+\left\vert s \right\vert}{T}{d\left( v,s \right)}^{2}+\frac{\left\vert v \right\vert+\left\vert t \right\vert}{T}{d\left( v,t \right)}^{2}-{\frac{\left\vert v \right\vert}{T}d\left( s,t \right)}^{2}}$ | Equation 3 |
| --- | --- |

Where $u$ is the newly joined cluster consisting of clusters $s$ and $t$, $v$ is an unused cluster in the forest, $T=\left| v \right|+\left| s \right|+\left| t \right|$.

2. Instructions for data processing

We built an automated data processing pipeline with edtools, which uses commands in DIALS or PETS2 in the backend. Before running edtools, make sure that all the t-SerialED datasets are put in one folder. We divide the whole workflow into 5 commands to give the user full control of the whole workflow: 1) edtools.update_dials, 2) edtools.autoindex, 3) edtools.extract_dials_info, 4) edtools.find_cell, and 5) edtools.dials_to_CrystFEL. Fully automated data processing can be achieved by putting these commands into one bash script. The data processing time for 902 datasets requires 6h (CPU: i7-5820K, 6 cores, 12 threads; RAM: 16G, DDR4; Win10).

**2.1 Preparation of data processing**

The direct output datasets from Instamatic program are in MRC format. We output SMV data format by specifying --write_smv True. The centers of diffraction patterns are aligned using cross-correlation. If the elliptical distortion is known, then it is also possible to specify the parameters of elliptical distortion and correct before the next step. For accurate unit cell indexing, PETS2 is needed and images in TIFF format are required. By specifying --write_tiff True, images in tiff format can be converted from MRC format.

Command: edtools.update_dials -cent True --write_smv True --write_tiff True --gain 7 --refine_center True --ellip_corr True

**2.2 Automatic Indexing**

For each dataset, edtools.autoindex_dials.exe will find the location of dials_process.bat file, in which dials.import, dials.find_spots and dials.index commands are included ([https://dials.github.io/ documentation/programs/dials_index.html](https://dials.github.io/%20documentation/programs/dials_index.html)). We used the default FFT3D indexing method of dials.index, which is often used to index rotational electron or X-ray datasets. This method uses a Fourier transform of all observed reciprocal space reflection positions to identify lattice periodicity. Potential basis vector directions are ranked, and typically those for the strongest lattice are found first. Once spots are indexed using that basis, some number of unindexed spots remain. The dials.index parameter max_lattice allows these spots to be recycled for another round of 3DFFT-based indexing, so that if max_lattice=2 a second lattice will be searched for, and if max_lattice=3 and a second lattice was already found, then the remaining unindexed spots will be recycled to look for a third lattice. If no such lattice is found, or the number of unindexed spots is low (by default less than 10% of the original number of spots) then the algorithm finishes with the number of lattices it found so far. Using a restrain file (Figure S 6), the users have the option to specify the space group, unit cell, geometry refinement parameters and multi-lattice searching parameters. If the space group and unit cell parameters in the restrain file are set to None, then DIALS will determine the space group and unit cell automatically. The indexing algorithm from DIALS can not only index single crystal datasets but also index multi-crystal datasets. As resolution decreases and number of reflections decreases, the ability to resolve the number of multi-crystals in one dataset decreases. Figure S7 shows typical datasets that are indexable. However, if there are too few reflections or the sample is polycrystalline, the indexing algorithm will fail (Figure S8)

Command: edtools.autoindex_dials.exe --restrain True

**2.3 Extract unit cell information**

After all the datasets are indexed, edtools.extract_dials_info.exe is used to summarize the result and generate a list of datasets that fits the requirements. The requirements can be if the dataset only contains one lattice or the minimum number of indexed reflections or the percentage of indexed reflections. The purpose of the restraints is evaluation of the quality of indexing and checking if the indexing is reliable. To our experience, if the indexed reflections reach more than 100 and more than 30% of reflections are successfully indexed, then the indexing result is reliable. The output of this command is a cells.yaml file.

command: edtools.extract_dials_info.exe -t_i 100 -t_p 0.3

**2.4 Unit-cell-based clustering for phase analysis**

After the creation of cells.yaml, it can be read by edtools.find_cells for unit cell clustering using dendrogram. The command will calculate the distance between the obtained unit cells and set up a threshold to cluster unit cells. After the command, it will summarize the result and calculate the mean value for each cluster. For each cluster, a yaml file will be created in which all the unit cells and corresponding datasets will be recorded.

command: edtools.find_cell.exe cells.yaml -c True

**2.5 Data integration and merging**

This command can convert SMV files into HDF5 files with metadata, which is required by diffracttem^3^ and CrystFEL^2^. Then diffracttem and CrystFEL can be used to further process the data. This command can also directly use dials.ssx_integrate to integrate all the datasets in cells.yaml and then merge all the frames as if they are individual crystals using xia2.ssx_reduce (<https://dials.github.io/ssx_processing_guide.html>). The output of the command is a Shelx HKL file and a Shelx INS file, which can be input into Shelxt for structure solution.

command: edtools.dials_to_crystfel.exe cells.yaml --merge True --space_group Pnma -t 0.3 -d 0.9 --split True --integrate True


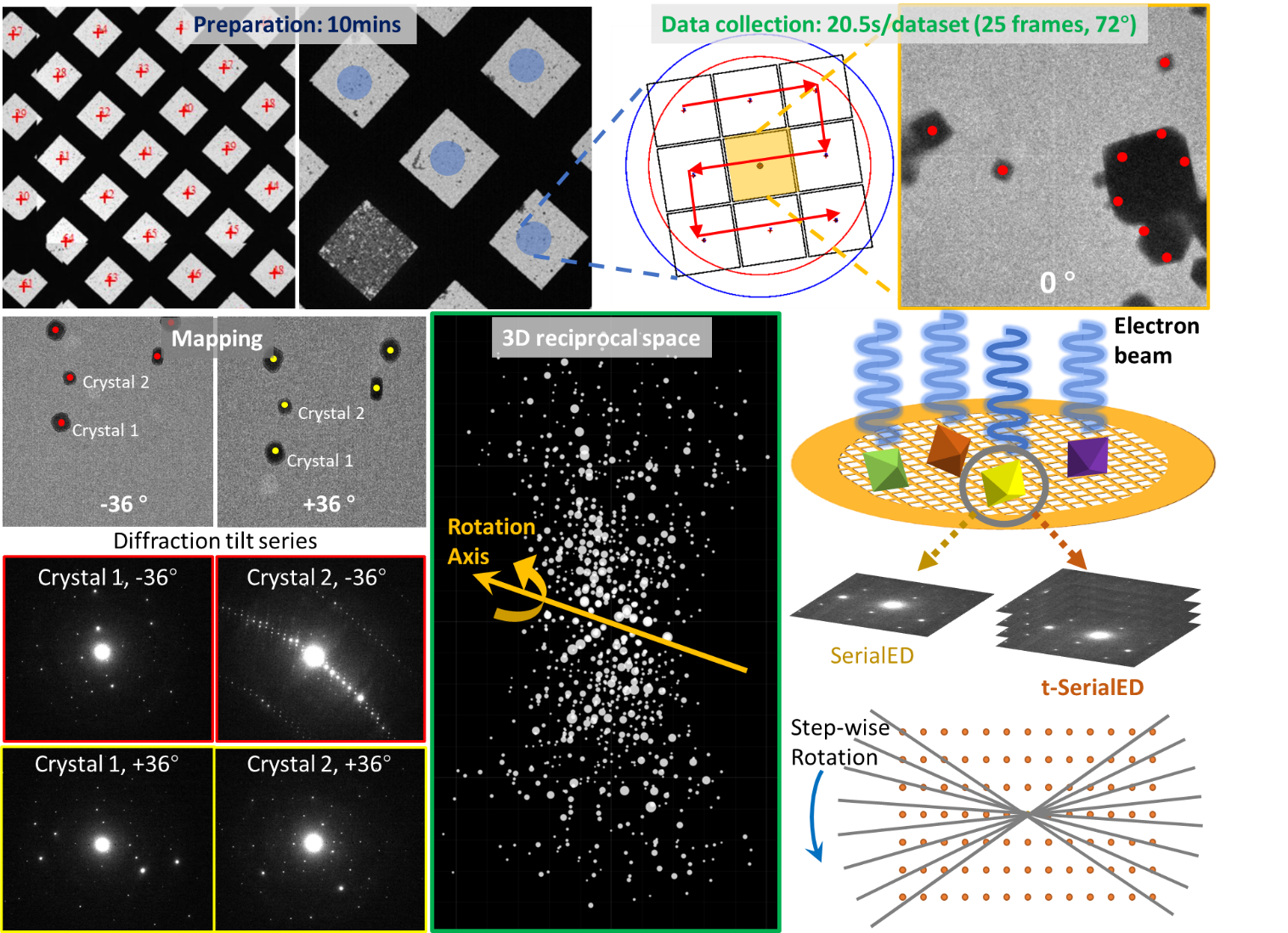


(a)

(b)

(c)

(d)

(e)

Figure S 1. Schematics of t-SerialED data acquisition process: (a) In the preparation stage, grid holes are found and eucentric height is determined under low-mag mode. (b) At the center of each grid hole, a user-defined area is divided into multiple patches and the stage will move to these patches. Within each patch, there are several crystals and discrete tilt series are collected batch-by-batch. (c) Crystals are identified automatically and ED patterns will be taken for each crystal. After all ED patterns are taken, the stage will tilt to another angle and repeat the same steps. The shift due to stage rotation is compensated by crystal tracking. (d) At last, the ED patterns taken at different tilt angles for each crystal can form a 3D reciprocal space, in which unit cell and orientation matrix can be reliably identified. (e) Comparison of SerialED and t-SerialED. SerialED captures only one snapshot from one crystal while t-SerialED collects multiple shots from different angles.


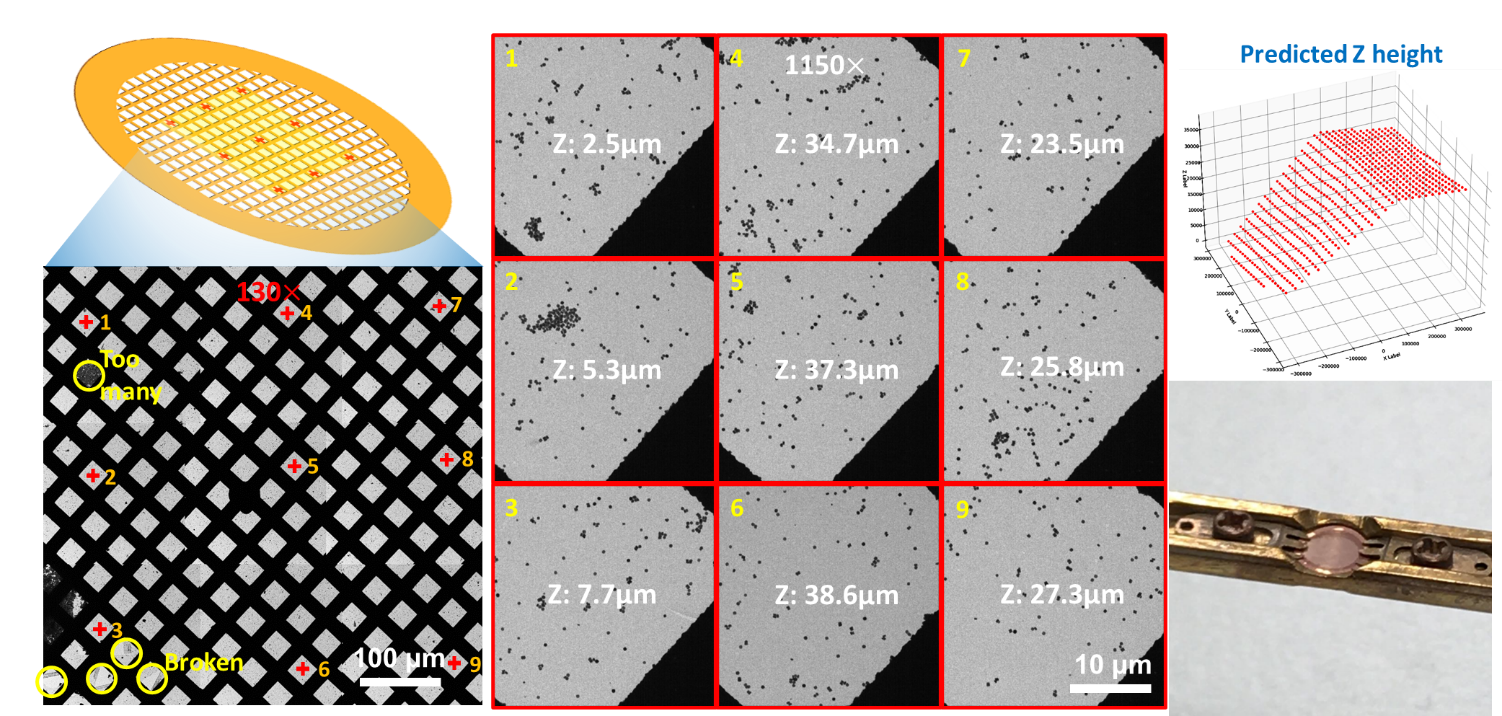


Figure S 2. Process for eucentric height prediction of the low-mag montage from measured eucentric heights at positions marked with ‘+’. The prediction plot shows the grid is curved and a photo of the loaded grid confirms the grid is indeed curved.


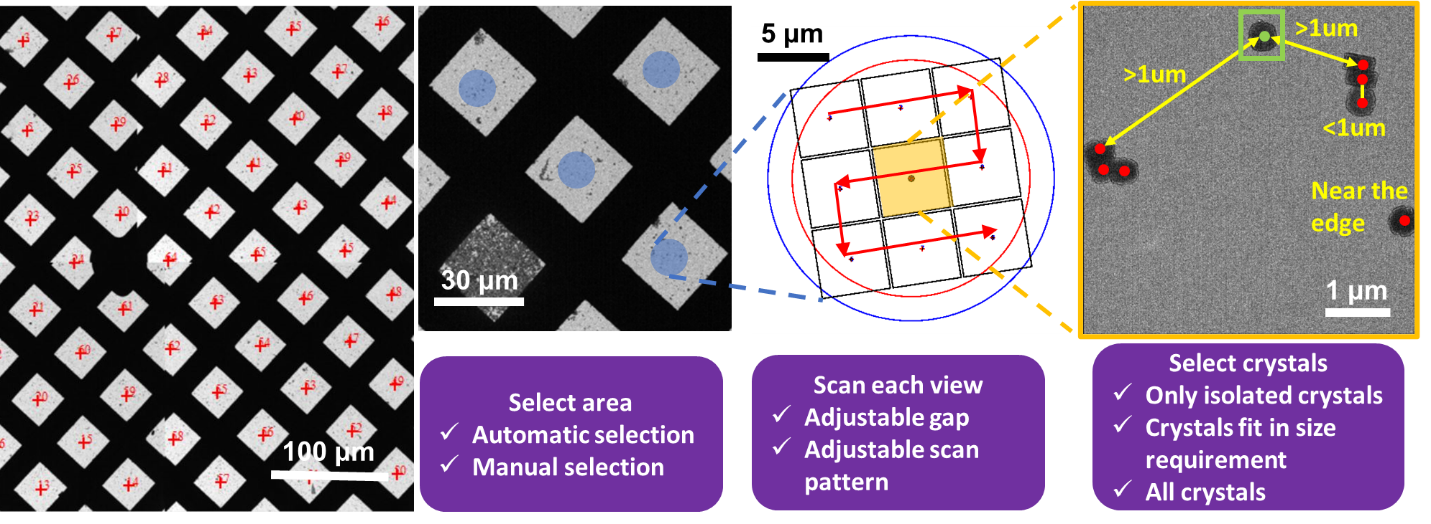


Figure S 3. Schematics of automatic crystal finding. The area selection procedure allows for both automatic selection and manual adjustment. The radius of the scan area can be determined by the user and it will be divided into smaller patches according to the desired magnification for crystal searching. The stage will move to the center of patches and take images and identify crystals at high magnification.


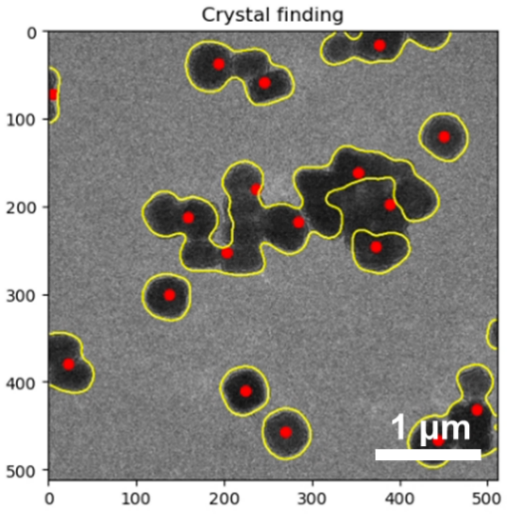

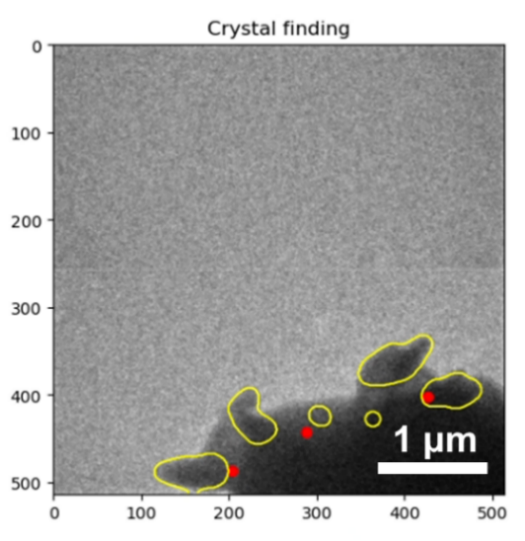

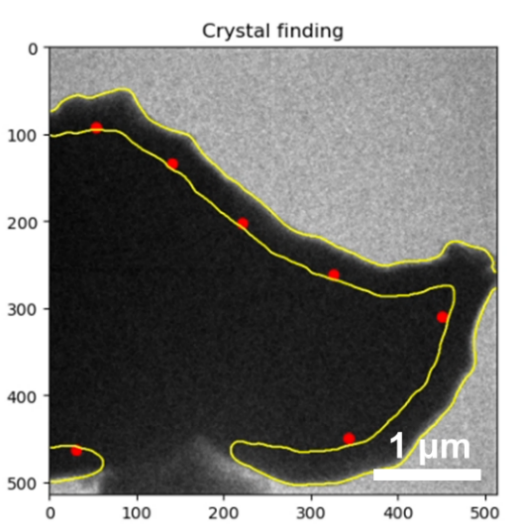

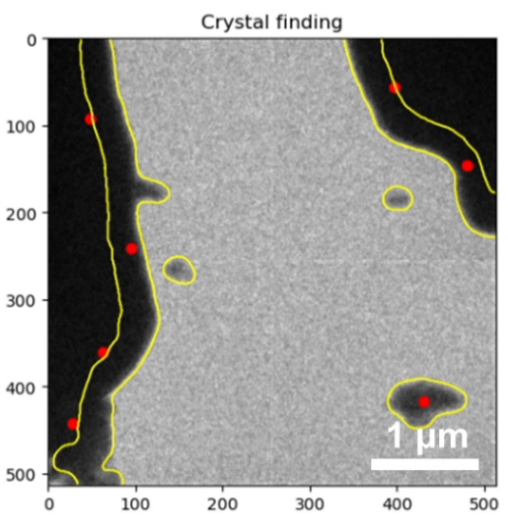


**(a)**

**(b)**

**(c)**

**(d)**

Figure S 4. Target picking for aggregated particles and large blocks. (a) For aggregated particles, the density of the target spots depends on the user-defined beam size. If the beam hits two or three particles at the same time, the indexing algorithm in DIALS is still able to distinguish each set of lattices. (b, c, d) For large blocks, the algorithm will identify the edge of a large block and put red target spots along the edge according to the user-defined density.


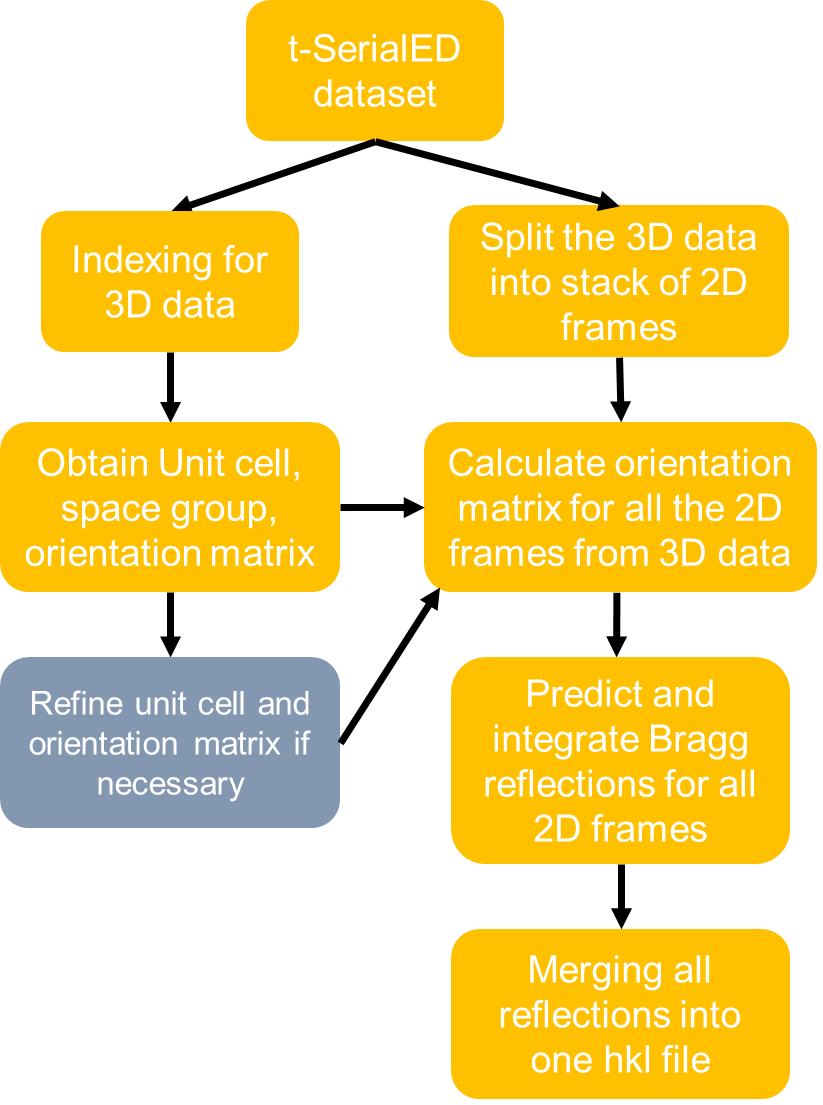


Figure S 5. Flowchart of data processing of t-SerialED datasets


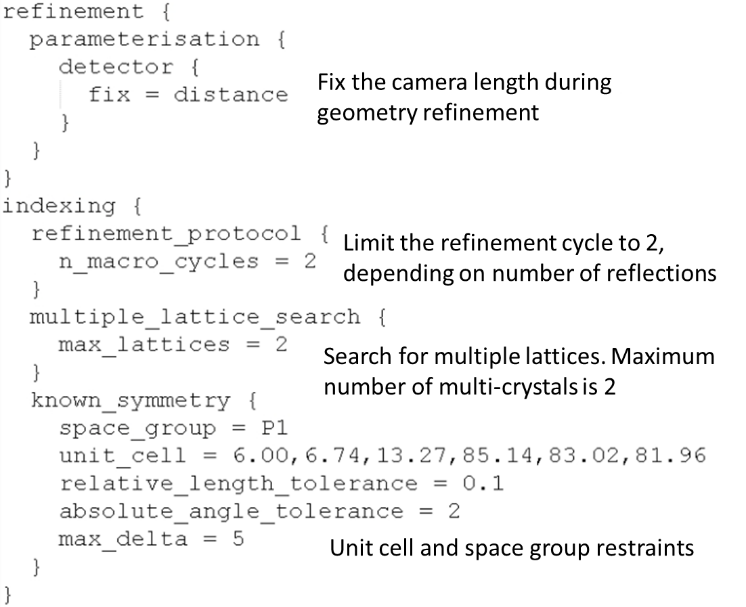


Figure S 6. An example of the restrain.phil file

**5 Å^-1^**


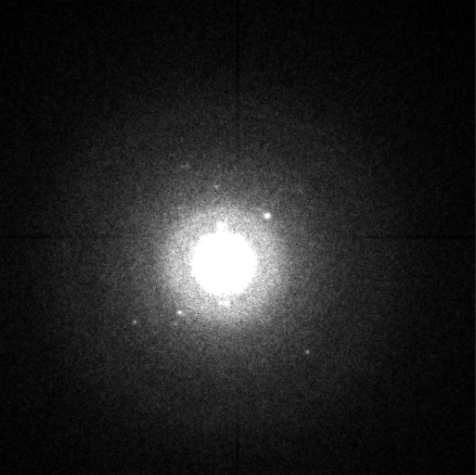

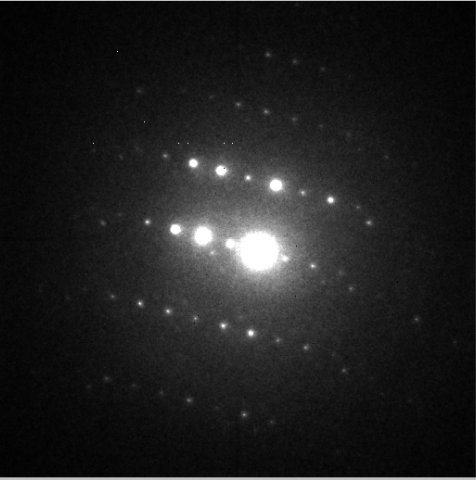

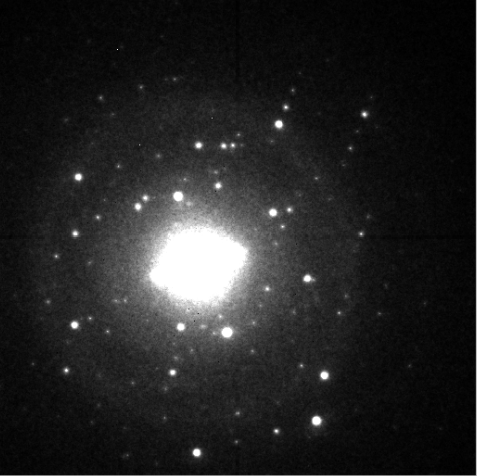

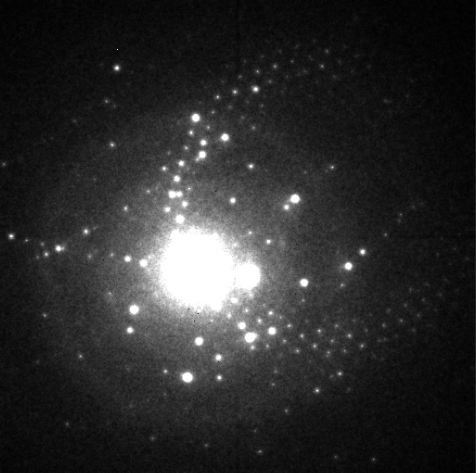


**(a)**

**(b)**

**(c)**

**(d)**

**5 Å^-1^**

**5 Å^-1^**

**5 Å^-1^**

Figure S 7. Typical t-SerialED datasets that can be indexed: (a) one crystal (b) two crystals (c) three crystals (d) low resolution, one crystal


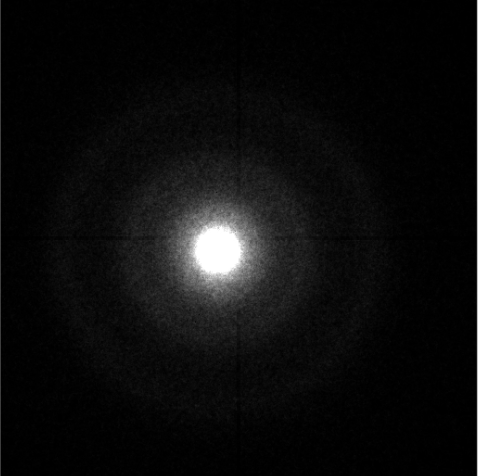

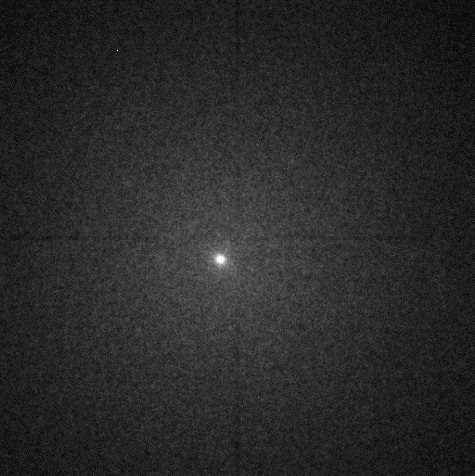

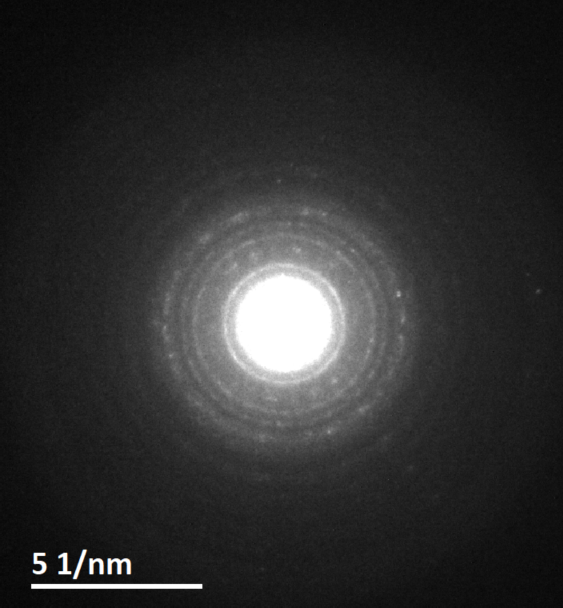


**(a)**

**(b)**

**(c)**

**5 Å^-1^**

**5 Å^-1^**

**5 Å^-1^**

Figure S 8. Typical t-SerialED datasets that are not indexable during data processing: (a) blocked (b) amorphous or blank (c) polycrystalline.


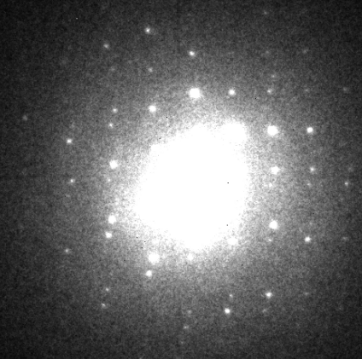

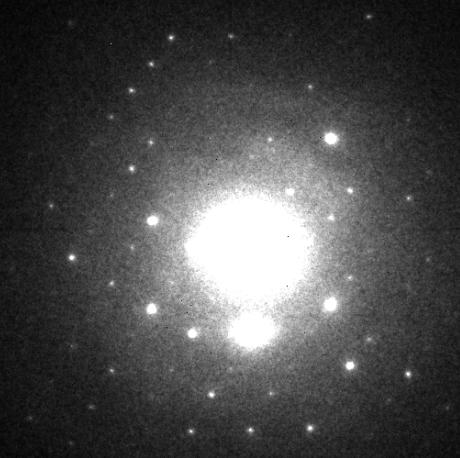

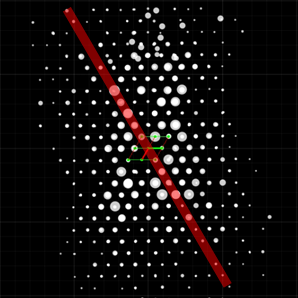

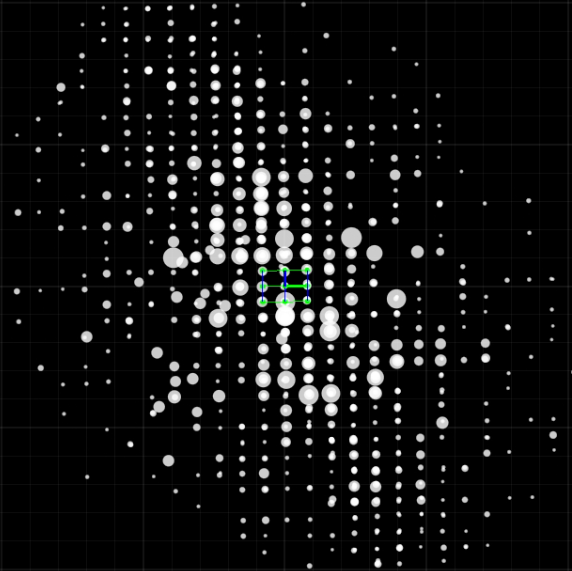

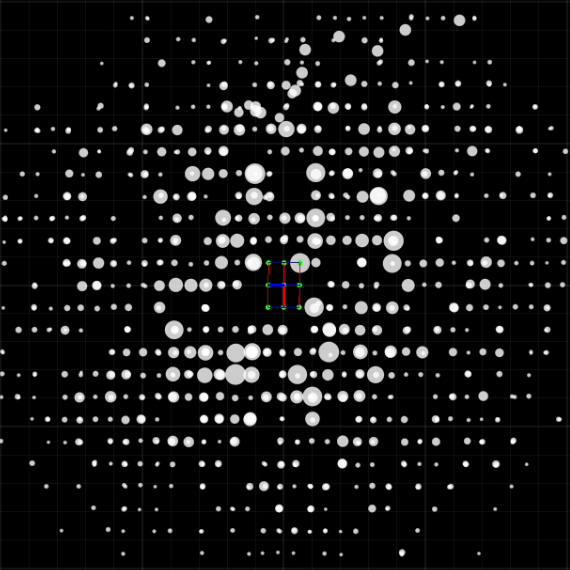

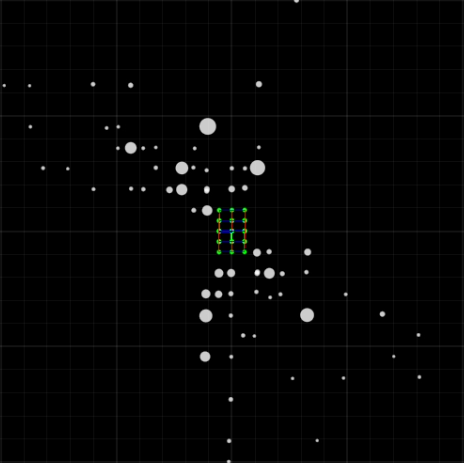


**(a)**

**(b)**

**(c)**

**(d)**


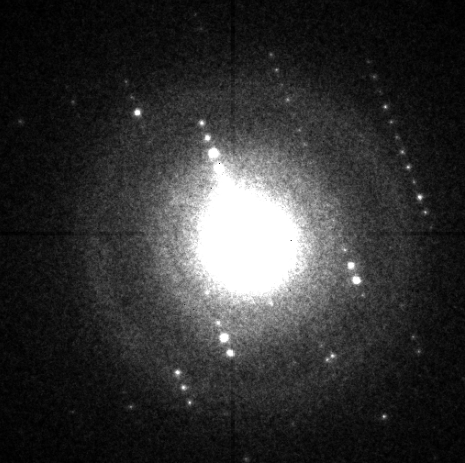

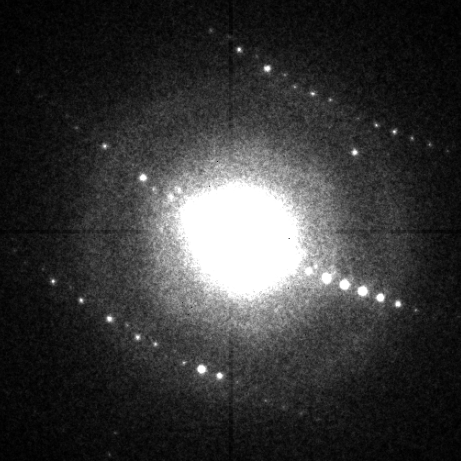


**(e)**

**(f)**

**(g)**

**(h)**

Figure S 9. Reconstructed 3D reciprocal space of an individual tilt series for MOF-235. Space group can be determined by checking the systematic absence of reflections. Reciprocal space viewed along: (a). a*-axis (b). b*-axis (c). c*-axis. (d). (hh-2hl) plane sliced from 3D reciprocal space in red. Tilt series of MOF-235: (e, f) start and end frame for one tilt series; (f, h) start and end frame for another tilt series.


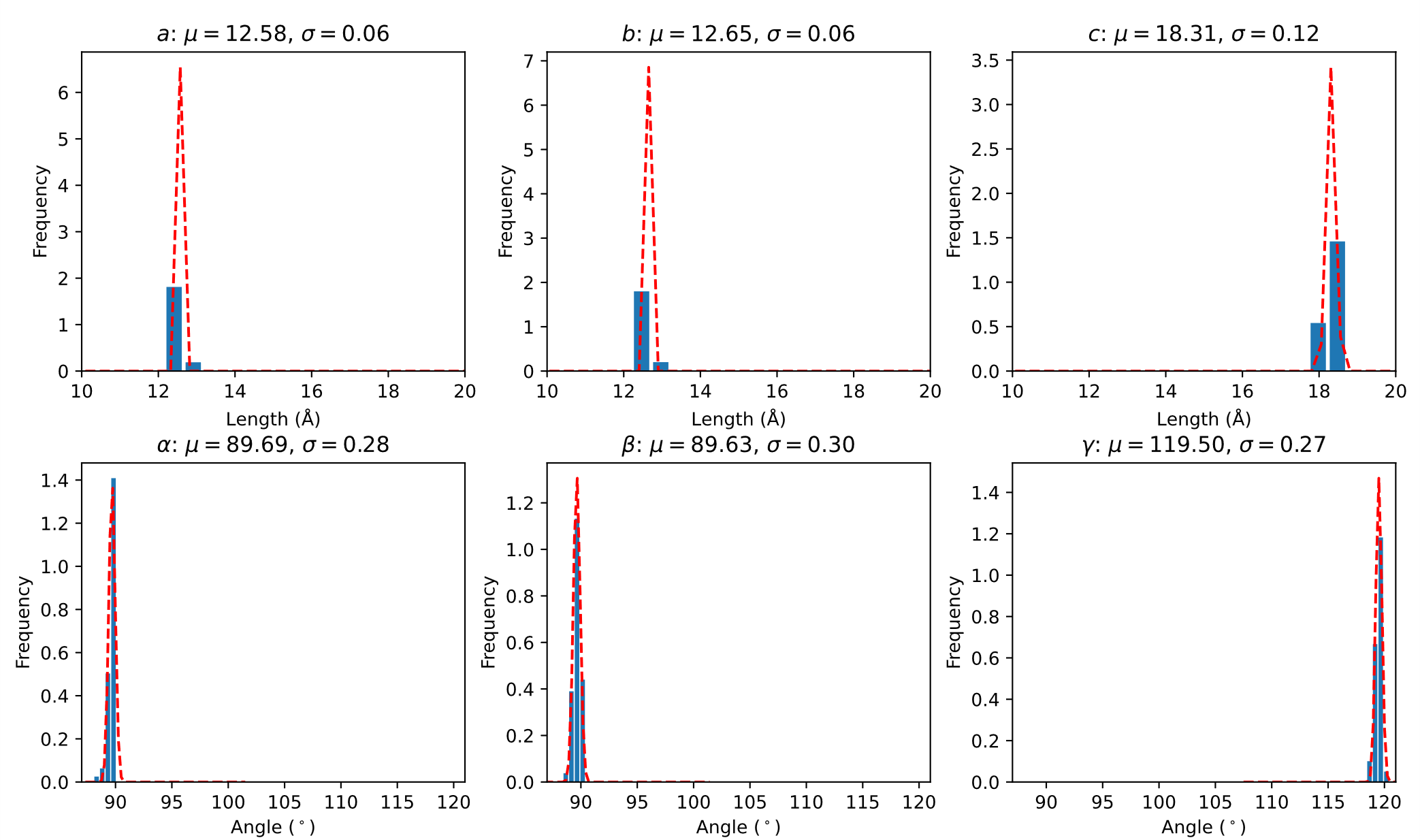


Figure S 10. Unit cell distribution analysis for MOF-235. The datasets are indexed with P1 to evaluate the accuracy of unit cell. The mean values and standard deviations of all axes and angles are listed.


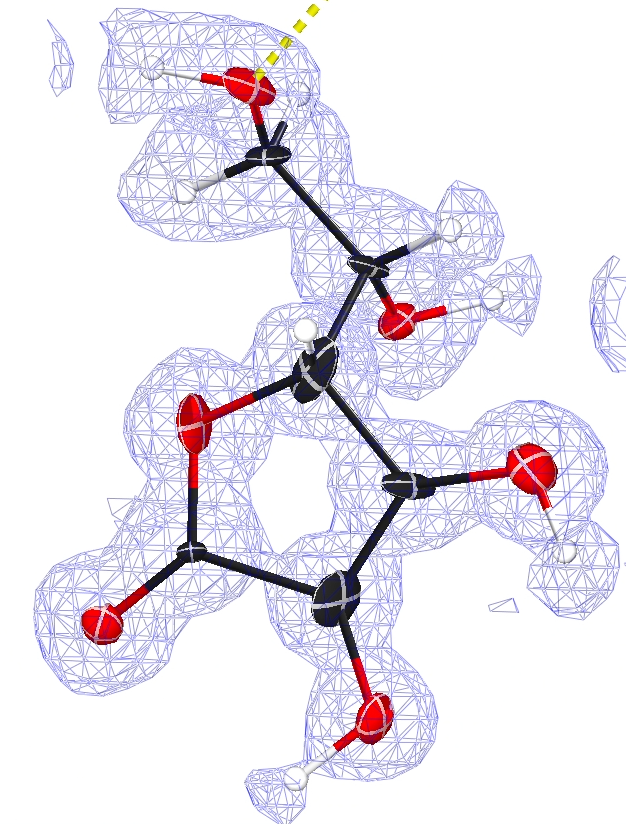

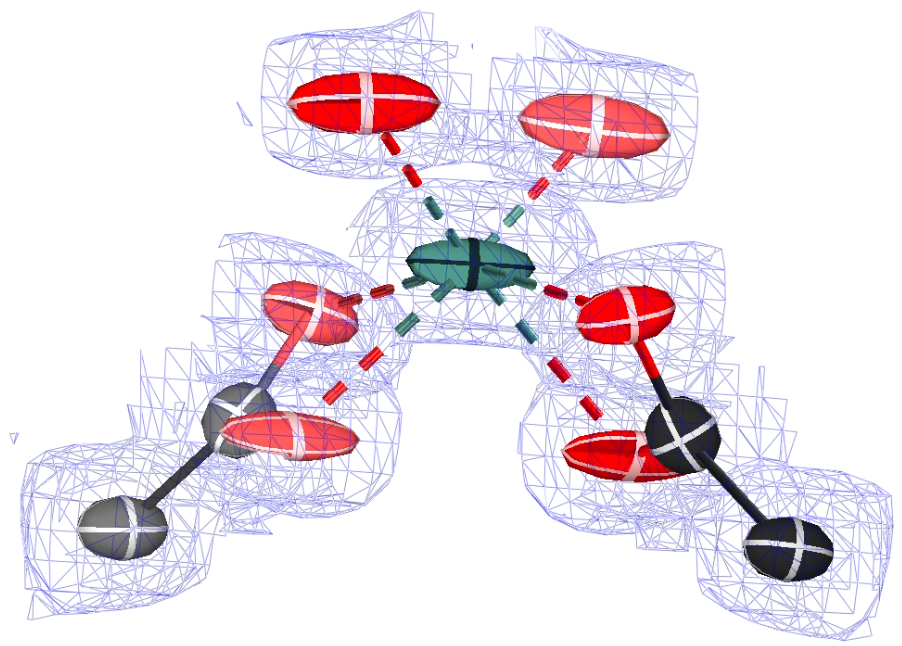

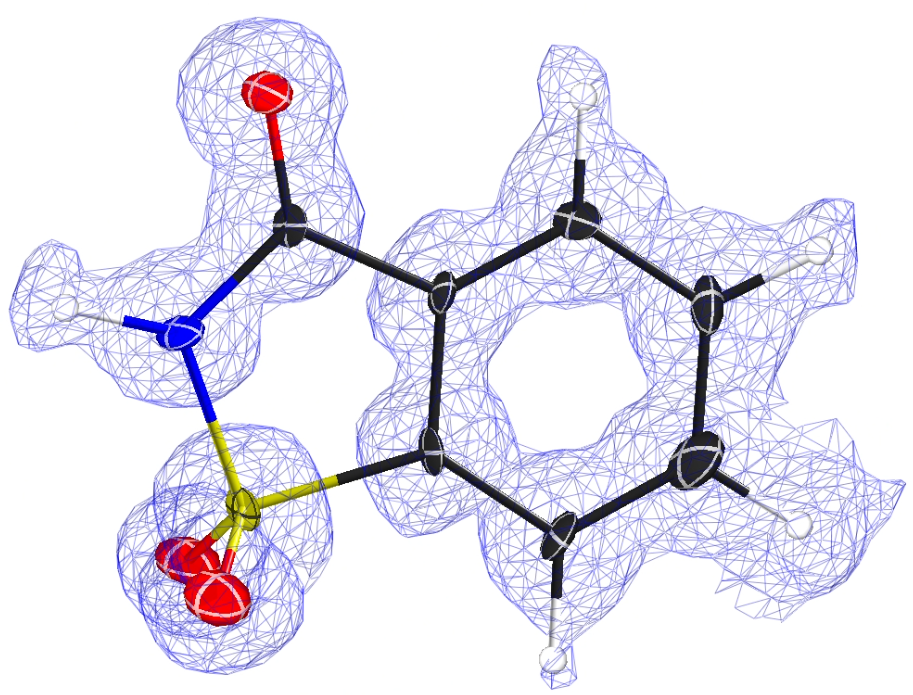

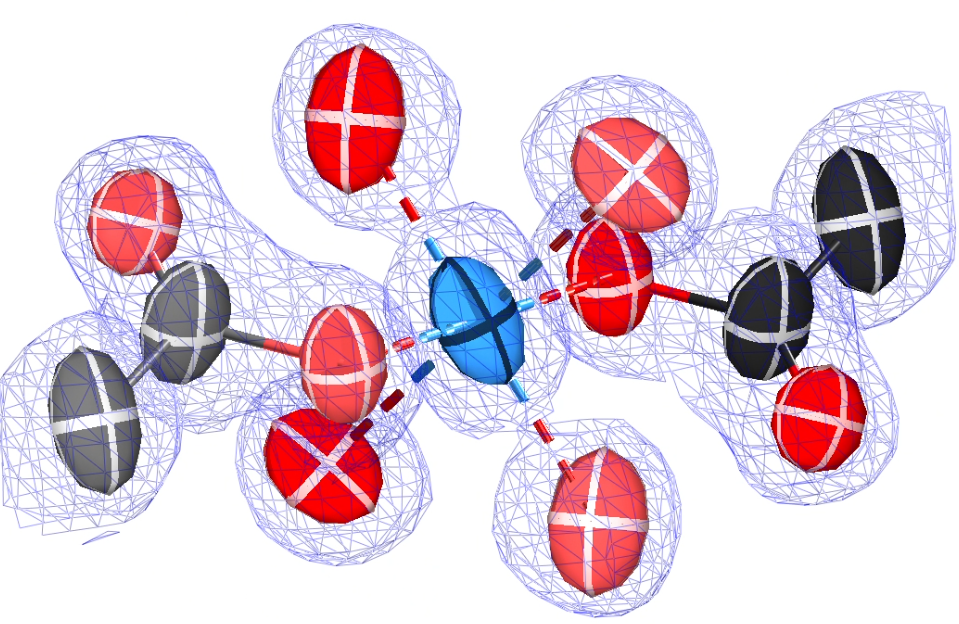


**(a)**

**(b)**

**(c)**

**(d)**

Figure S 11. Structure determination results for the complex mixtures: structures and electron potential maps for (a). L-ascorbic acid; (b). Zn acetate; (c). Saccharin; (d). Mg acetate.


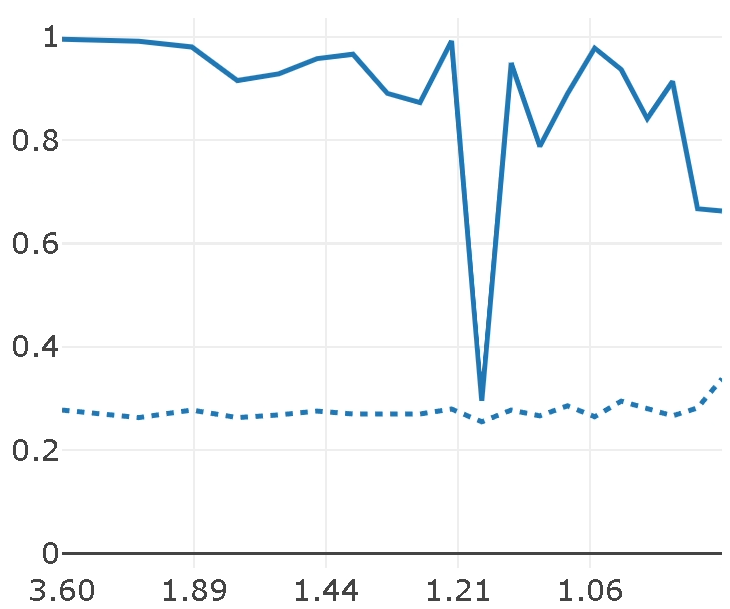

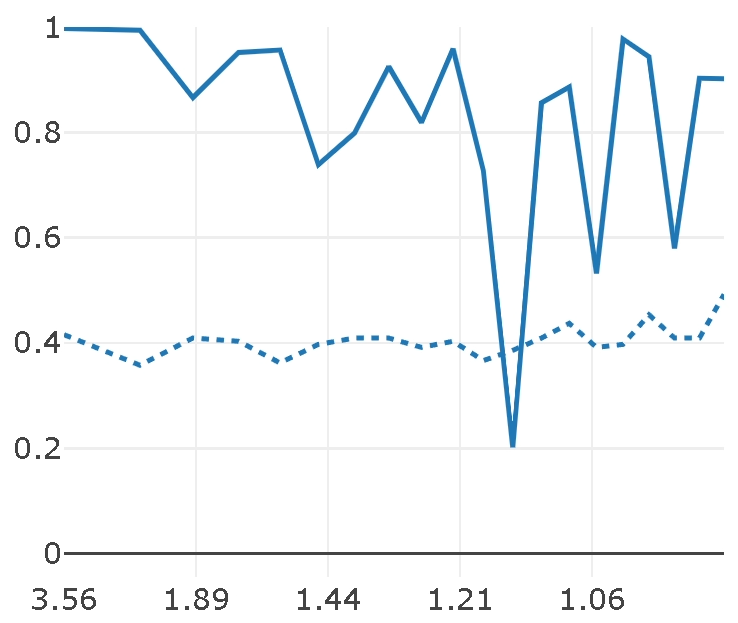

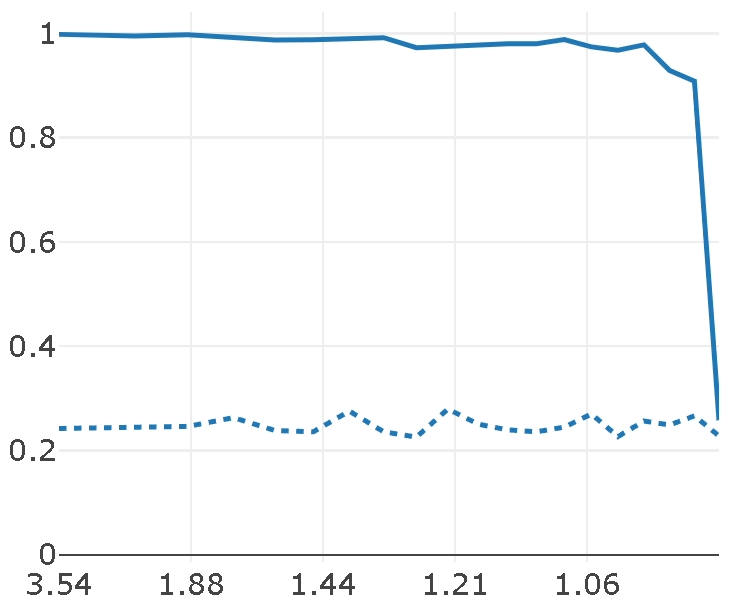

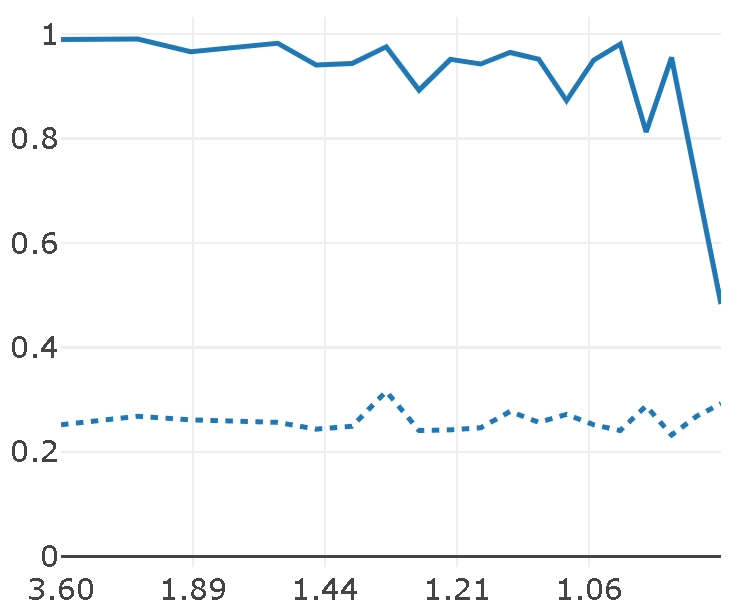

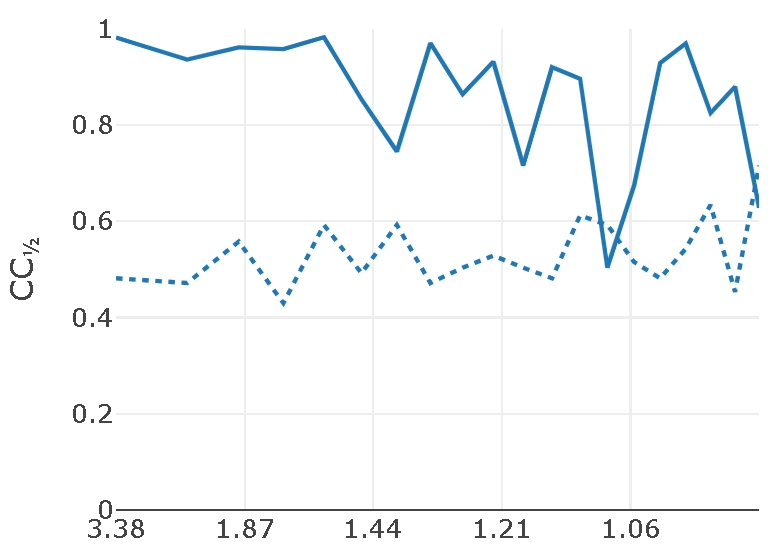

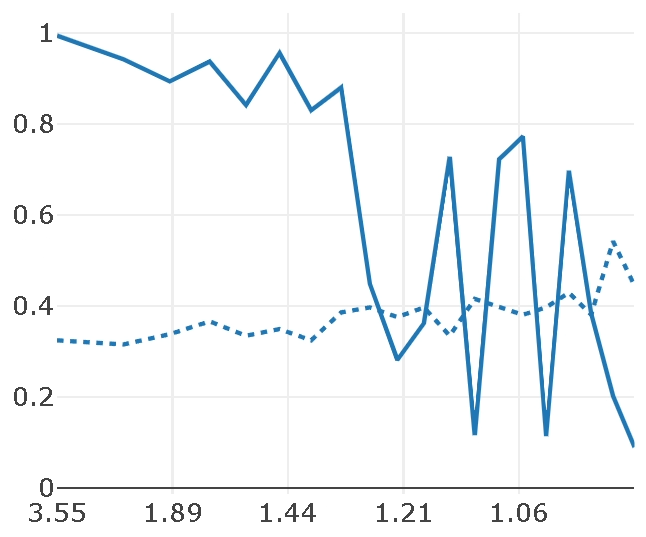

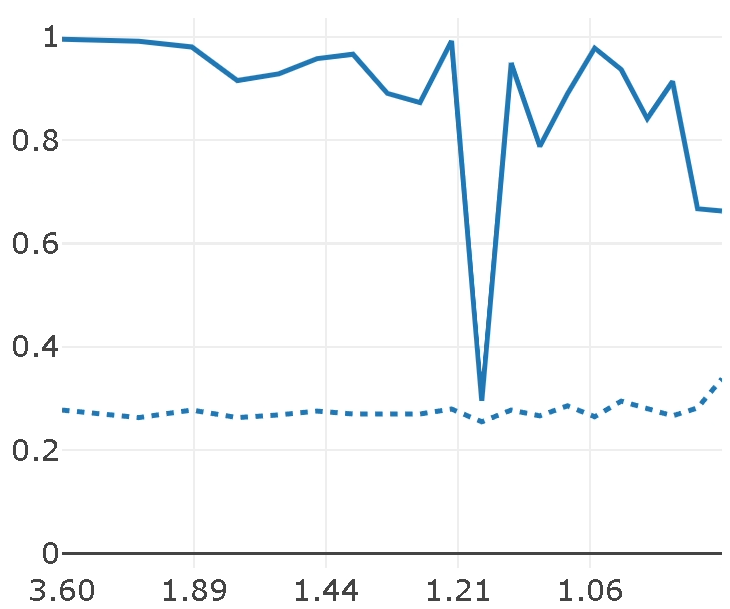


**(a)**

**(b)**

**(c)**

**(d)**

**(e)**

**(f)**

Resolution (Å)

Resolution (Å)

Resolution (Å)

Resolution (Å)

Resolution (Å)

Resolution (Å)

*CC_1/2_*

*CC_1/2_*

*CC_1/2_*

*CC_1/2_*

*CC_1/2_*

*CC_1/2_*

Figure S 12. *CC_1/2_* plot of (a) Glycine (b) L-ascorbic acid (c) Zn acetate (d) Saccharin (e) Mg acetate (f) L-glutamic acid. The dashed lines represent the CC_1/2_ values that are considered statistically significant at different resolutions.


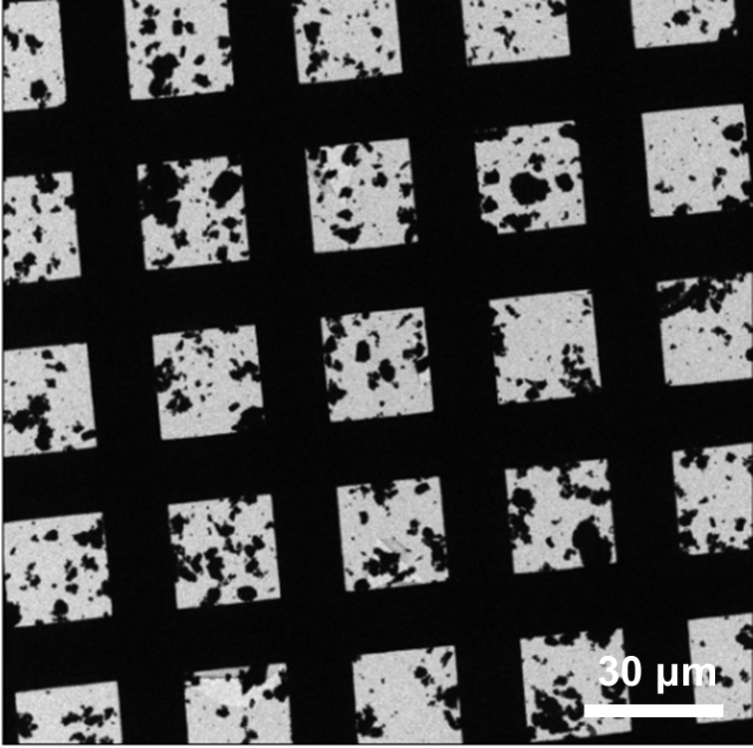

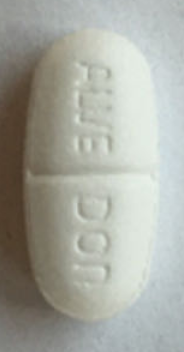

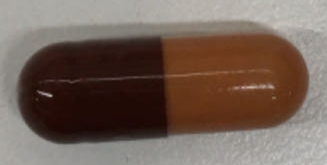

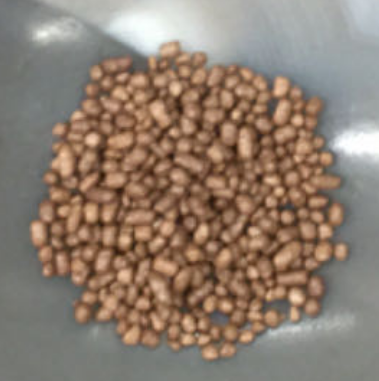


**(a)**

**(b)**

**(c)**

**(d)**

Figure S 13. Photos of the (a) tablet, (b) capsule and (c) pellet. (d) The overall morphology of the Fe Supplement sample. After crushed in mortar, the shapes of the ingredients are irregular.


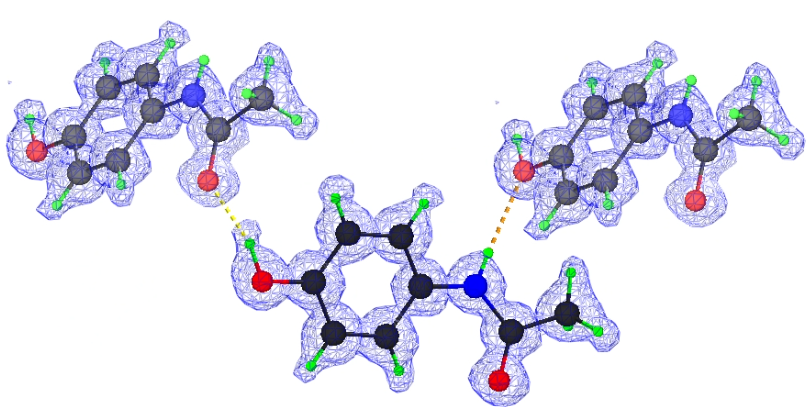

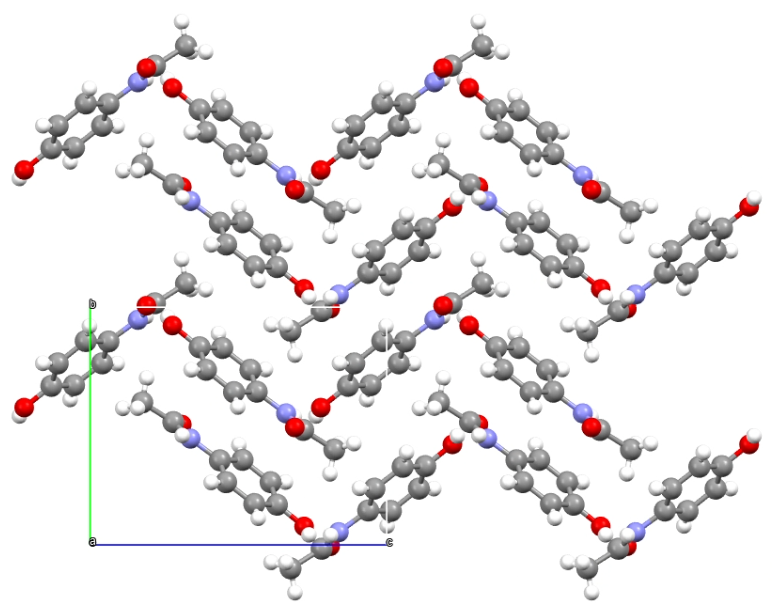

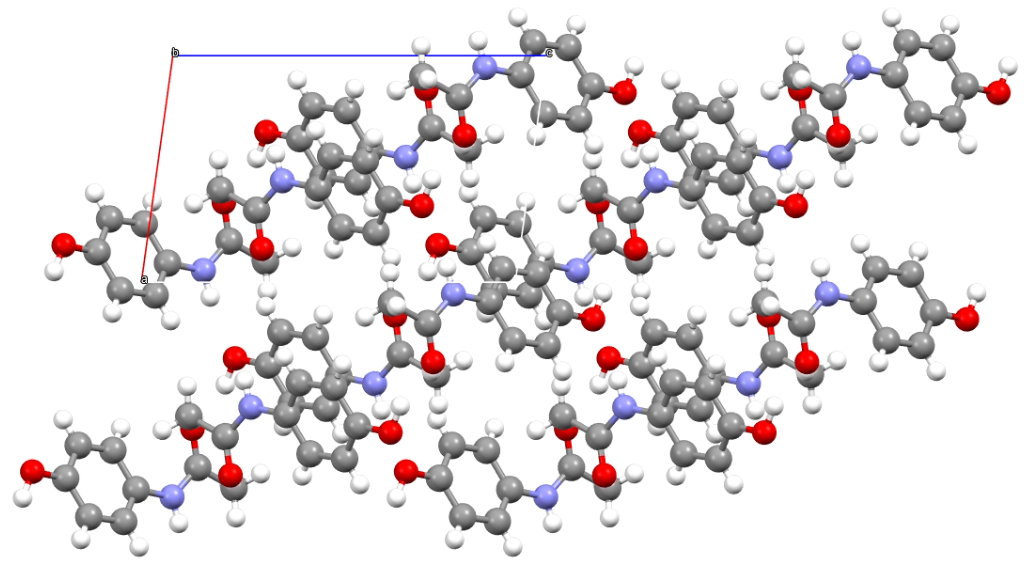


**(a)**

**(b)**

**(c)**


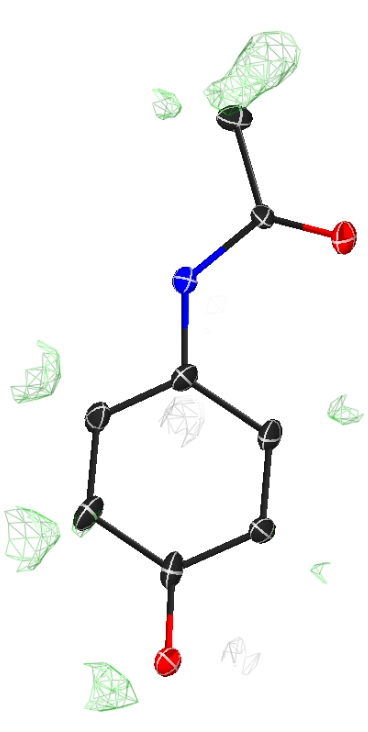


**(d)**

Figure S 14. (a, b). Crystal packing of paracetamol, (c) hydrogen bonding network in the crystal and electron static potential map for the molecule. The green atoms in (c) are hydrogen atoms and the yellow dashed lines are hydrogen bonds. (d). Residue map of the paracetamol molecule proves the existence of hydrogen atoms.


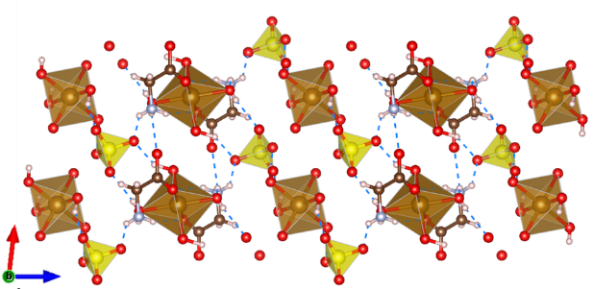

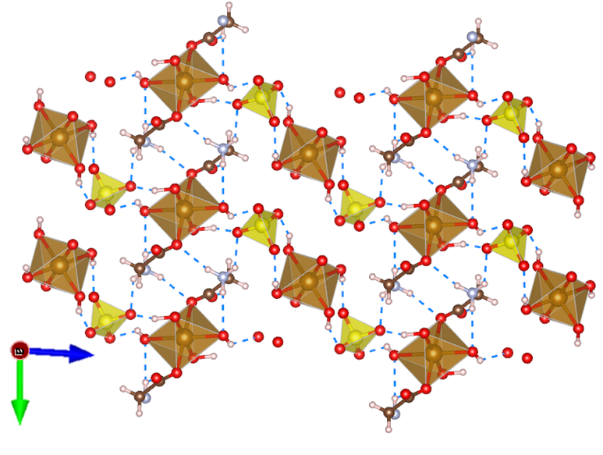

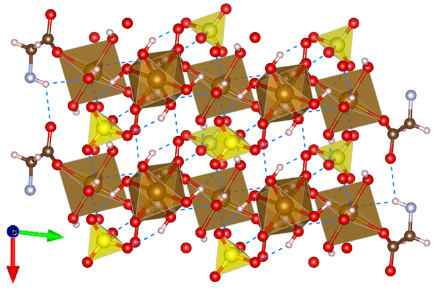


**(a)**

**(b)**

**(c)**

Figure S 15. Atomic arrangement of ferrous glycine complex viewed from different directions. The dashed lines represent the hydrogen bonding in the crystal.


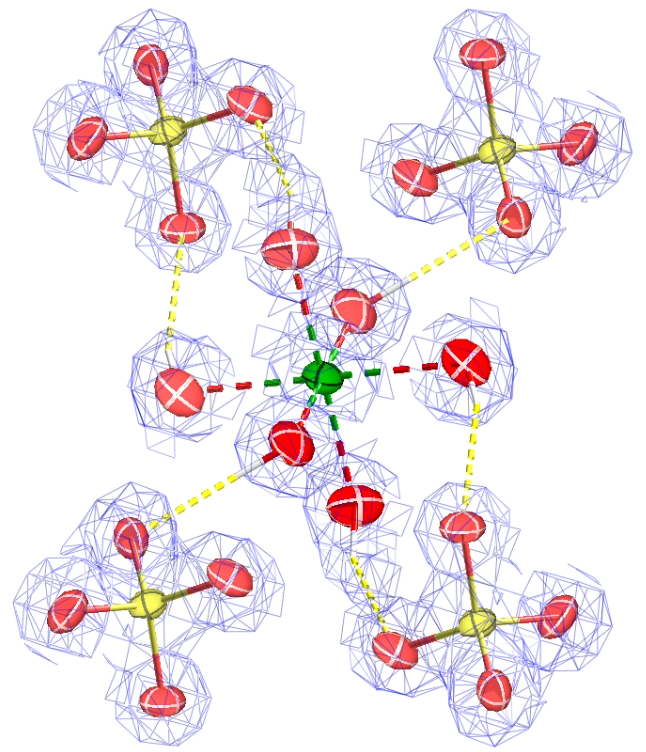

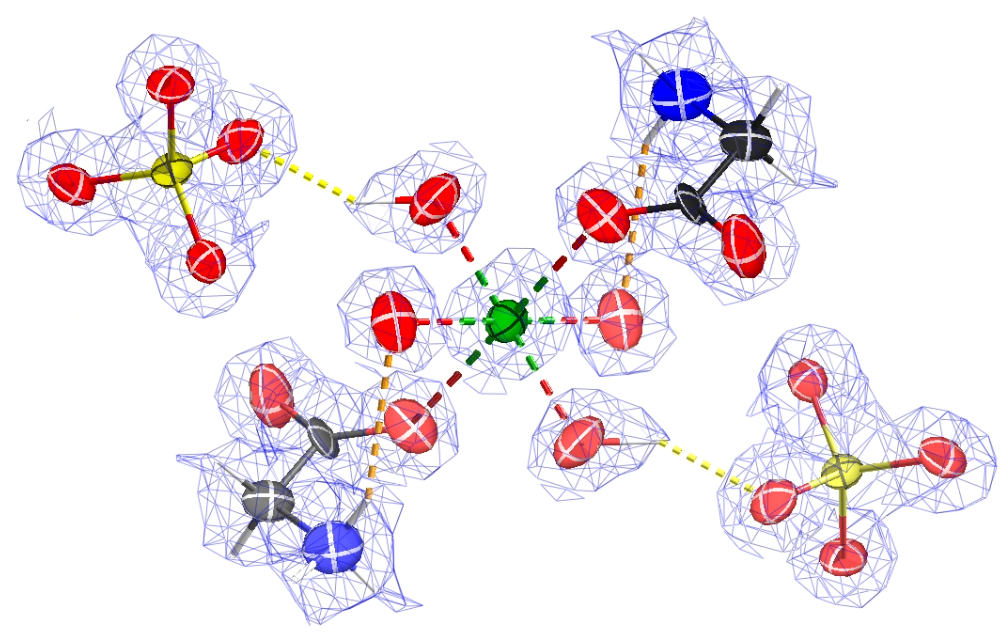


**(a)**

**(b)**

Figure S 16. Coordination environment of two independent Fe atoms


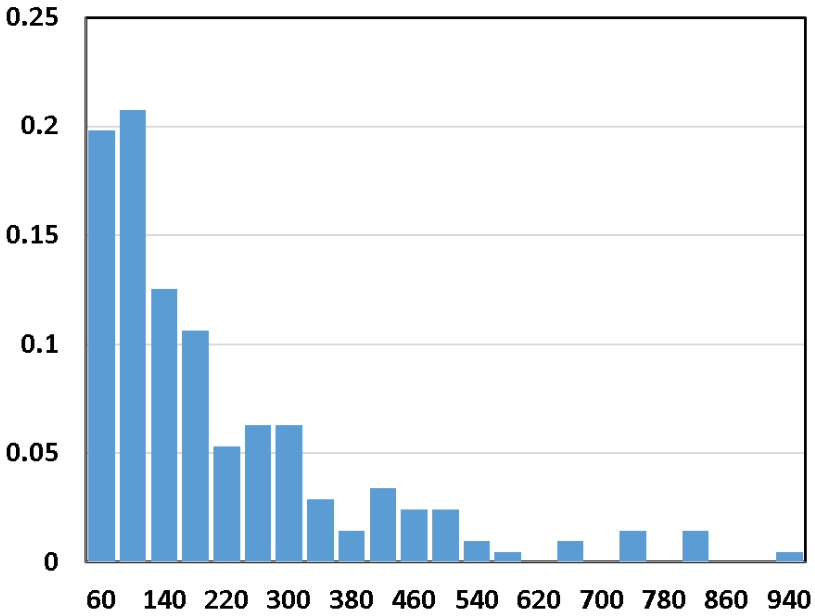

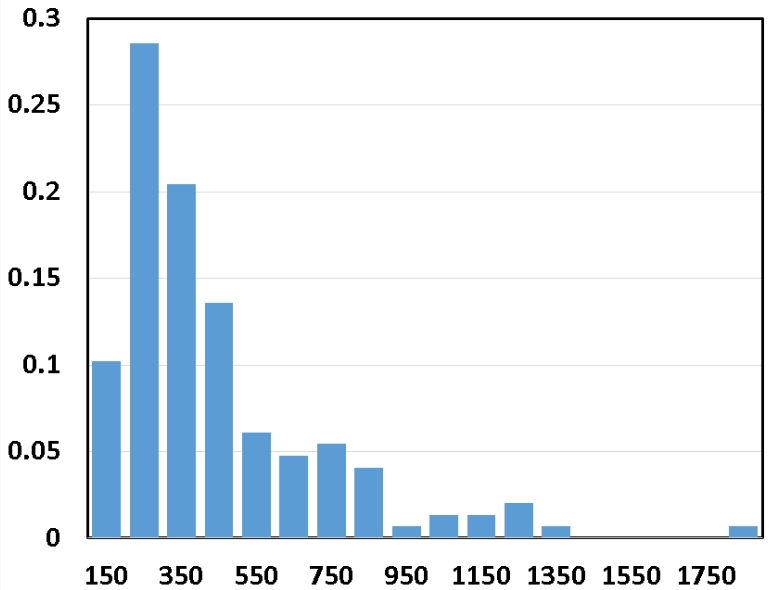


Number of spots

Number of spots

Ratio

Ratio

Figure S 17. Histogram of number of indexed reflections. (a) Paracetamol (b) Fe supplement. Horizontal label represents the number of indexed reflections while vertical label is the frequency. The median number of indexed reflections for paracetamol and Fe supplement is around 340 and 140, respectively

2 theta (°)


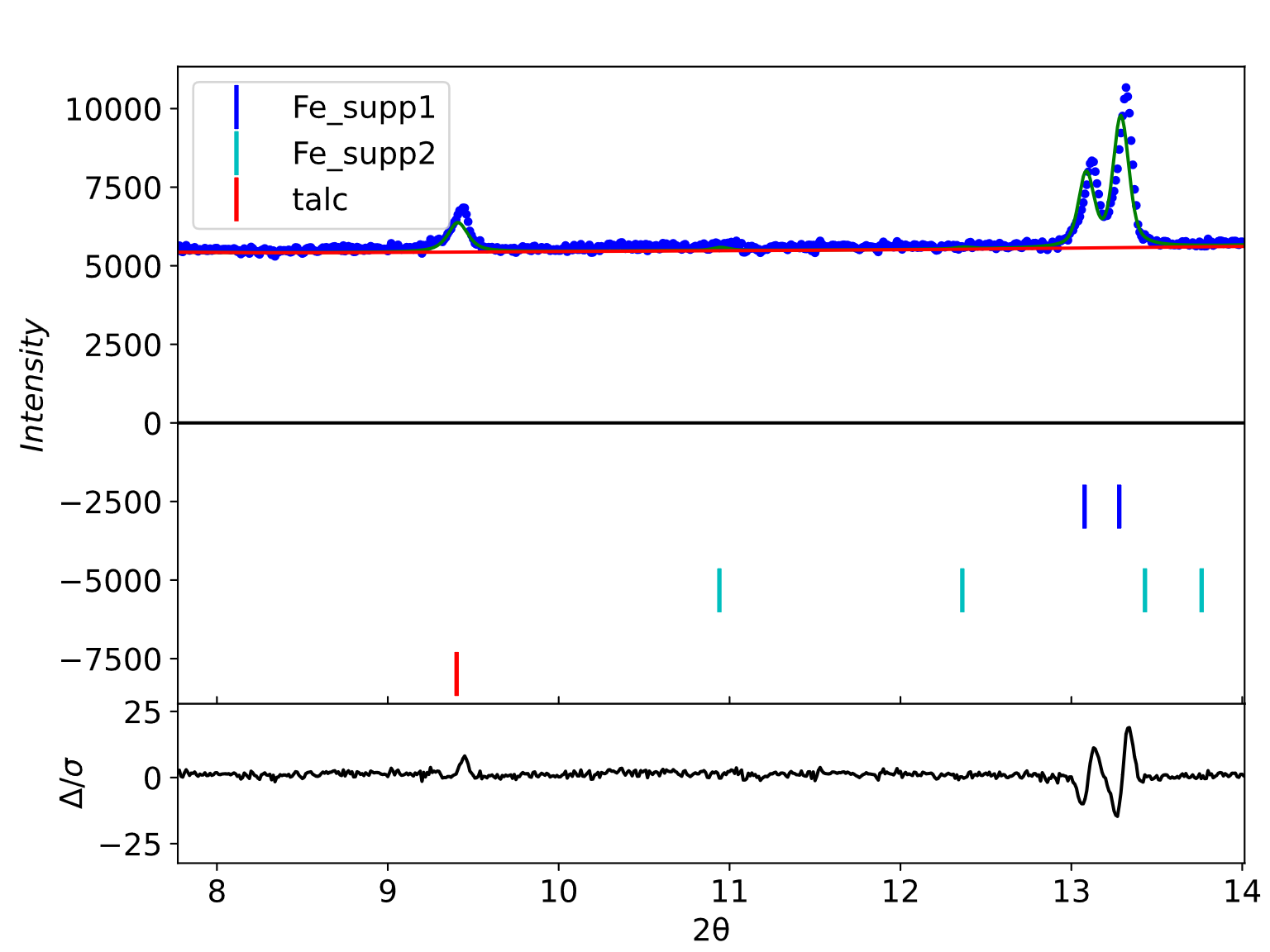

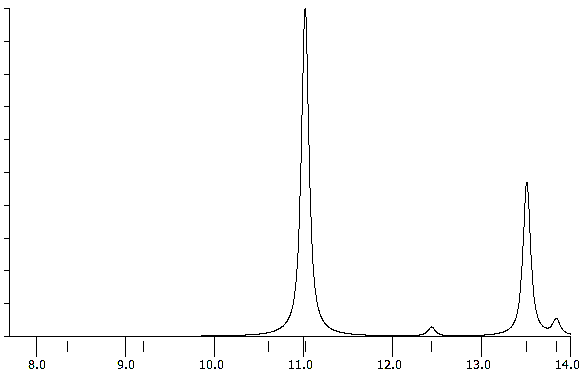


**(a)**

**(b)**

2 theta (°)

Figure S 18. Fe supplement: (a) Magnified Rietveld refinement results within 2θ range of 8° to 14°. (b) Simulated PXRD for the Fe_supp2 phase within 2θ range of 8° to 14°, showing the peaks located at 10.9° and 13.4° are the strong peaks. No visible peaks from Fe_supp2 can be found in (a), proving this phase is invisible in PXRD.

Table S 1. Setups and time required for preparation stage of the data collection.

|  | Low-magnification montage | *Medium-magnification image (1 × 1 tile) | Preparation Stage (mins) | | Total time （h） | # of datasets |
| --- | --- | --- | --- | --- | --- | --- |
| MOF-235 | 1 × 1 tile | 4 | | ~10 | ~1 h | 160 |
| Complex mixtures | 3 × 3 tile | 16 | | ~70 | ~6.5h | 902 |
| Paracetamol | 2 × 2 tile | 9 | | ~35 | ~1.7h | 197 |
| Fe glycine complex | 3 × 3 tile | 9 | | ~50 | ~3.8h | 496 |

* Eucentric height prediction

Table S 2. Statistics from data acquisition for MOF-235 and the mixture. The unindexable datasets include typical amorphous ED patterns, blocked ED patterns and polycrystalline ED patterns. The data collect time excluded the time spent for the prepration stage.

|  | MOF-235 | Complex Mixtures |
| --- | --- | --- |
| No. of datasets collected | 160 | 902 |
| Data collection time (h) | 1 | 6.5 |
| No. of unindexable datasets | 1 | 407 |
| No. of indexed datasets | 159 | 495 |

Table S 3. Ingredient composition in paracetamol tablet and Fe supplement capsule. The unindexed datasets include typical amorphous ED patterns, blocked ED patterns and polycrystalline ED patterns. The data collect time excluded the time spent for the prepration stage.

|  | Paracetamol tablet | Fe supplement capsule |
| --- | --- | --- |
| API compound | Paracetamol | Ferrous (II) glycine sulfate complex |
| API weight (mg) | 500 | 567 |
| Total weight (mg) | 590 | 703 |
| Density (API, g/cm^3^) | 1.29 | 1.98 |
| Density (tablet/capsule, g/cm^3^) * | 1.24 | 1.22 |
| Expected API weight ratio | 84.7% | 80.6% |
| Expected API volume ratio | 81.4% | 49.6% |
| Data collection time (h) | 1.7 | 3.8 |
| No. of datasets collected | 197 | 496 |
| No. of unindexed datasets | 33 | 288 |
| No. of indexed datasets | 164 | 216 |
| No. of datasets in API unit cells | 163 | 215 (207 form 1 + 8 form 2) |
| Measure API volume ratio (ED) | 82.8% | 43.4% |
| Non-API contents ** | Corn starch, pregelatinized starch (corn), povidone, potassium sorbate, **talc**, stearic acid, hypromellose, triacetin. | Ascorbic acid, microcrystalline cellulose, hypromellose, hydro-xypropylcellulose, methacrylic acid-ethyl acrylate copolymer (1:1) dispersion 30% (Eudragit L30 D-55) (contains methacrylic acid-ethyl acrylate copolymer (1:1), sodium lauryl sulfate, polysorbate 80), acetyl-triethyl citrate, **talc** |

* Measured using drainage method

** Composition was obtained from the datasheets from the official website of Swedish Medical Products Agency

Table S 4. Summary for acquisition time and accumulated dose for different collection frames. The overhead time is the extra time required when the detector is not acquiring frames, including the time for locating crystals, adjusting eucentric height, switching states, rotating the stage, moving the beam, and translating the stage.

|  | Time for ED frames (s) | Time for imaging frames (s) | Total overhead time (s) | Dose for imaging and overhead (e/ Å^2^) | Total time (s) | Total dose (e/ Å^2^) |
| --- | --- | --- | --- | --- | --- | --- |
| 7 frames (fast) | 3.5 | 1.8 (7 images) | 2 | 0.019 | 7.3s | 0.544 |
| **25 frames (medium)** | **12.5** | **3 (12 images)** | **5** | **0.040** | **20.5** | **1.915** |
| 49 frames (slow) | 24.5 | 3 (12 images) | 9 | 0.060 | 36.5 | 3.735 |

Table S 5. Comparison of the throughput of t-SerialED with previously reported methods based on electron diffraction. Note that the speed calculation does not include the time for preparing the experiment.

| Method type | Microscope | Speed (s/crystal) | Crystal Picking | Average tilt angle (°) | References |
| --- | --- | --- | --- | --- | --- |
| **t-SerialED (25 frames)** | **Themis-Z** | **20.5** | **Automatic** | **72** | **This work** |
| SerialED | JEOL-2100 | 2 | Automatic | 0 | ^4^ |
| SerialED | FEI Tecnai | 1.36 | Automatic | 0 | ^2^ |
| SerialED | Themis-Z | 1.5 | Manual | 0 | ^5^ |
| 3DED | JEOL-2100 | 60 | Automatic | 16 | ^6^ |
| 3DED | JEOL-2100 | 67 | Automatic | 20 | ^7^ |
| 3DED | Titan Krios | 648 | Manual | 80 | ^8^ |
| 3DED | JEM-2100/ ARM300 | >150 | Manual | ~130 | ^9^ |
| 3DED | Talos Arctica | >60 | Manual | 50 | ^10^ |

Table S 6. Data processing statistics of MOF-235 using CrystFEL

| d/A | nref | Possible | Completeness | Measured | Redundancy | Mean | CC | CC* | *R_split_*/% |
| --- | --- | --- | --- | --- | --- | --- | --- | --- | --- |
| 3.67 | 102 | 104 | 98.08% | 7533 | 73.9 | 4286.29 | 1 | 1 | 19.28 |
| 1.72 | 81 | 83 | 97.59% | 4272 | 52.7 | 7096.83 | 0.77 | 0.93 | 26.98 |
| 1.44 | 79 | 81 | 97.53% | 3422 | 43.3 | 4637.18 | 0.85 | 0.96 | 22.39 |
| 1.28 | 77 | 78 | 98.72% | 3103 | 40.3 | 2751.71 | 0.91 | 0.98 | 19.28 |
| 1.18 | 73 | 75 | 97.33% | 2577 | 35.3 | 2113.21 | 0.87 | 0.96 | 22.42 |
| 1.1 | 74 | 75 | 98.67% | 2389 | 32.3 | 1639.33 | 0.91 | 0.98 | 20.75 |
| 1.04 | 72 | 72 | 100% | 2123 | 29.5 | 1113.29 | 0.93 | 0.98 | 19.49 |
| 0.99 | 72 | 74 | 97.3% | 1828 | 25.4 | 959.93 | 0.76 | 0.93 | 27.76 |
| 0.95 | 74 | 75 | 98.67% | 1837 | 24.8 | 809.47 | 0.87 | 0.96 | 22.92 |
| 0.92 | 72 | 73 | 98.63% | 1595 | 22.2 | 681.9 | 0.86 | 0.96 | 20.86 |

Table S 7. Data processing statistics of MOF-235 using DIALS. Overall, low and high represent resolution shells.

|  | Overall | Low | High |
| --- | --- | --- | --- |
| High resolution limit | 0.90 | 2.44 | 0.90 |
| Low resolution limit | 18.36 | 18.36 | 0.92 |
| Completeness | 100.0% | 100.0% | 100.0% |
| Redundancy | 133.9 | 223.6 | 64.5 |
| I/sigma | 7.9 | 23.1 | 1.5 |
| R_merge_ | 1.061 | 1.619 | 1.186 |
| R_meas_ | 1.064 | 1.624 | 1.194 |
| R_pim_ | 0.081 | 0.106 | 0.132 |
| R_split_ | 0.194 | 0.169 | 0.641 |
| CC half | 0.989 | 0.994 | 0.533 |
| Total observations | 107404 | 12967 | 2708 |
| Total unique | 802 | 58 | 42 |

Table S 8 Data refinement statistics for MOF-235 datasets processed by CrystFEL and DIALS. The R_int_ in the dataset processed by CrystFEL is unknown because CrystFEL outputs a merged SHELX HKL files.

|  | **CrystFEL** | **DIALS** |
| --- | --- | --- |
| **Crystal system** | hexagonal | hexagonal |
| **Space group** | $P\overline{6}2c$ (190) | $P\overline{6}2c$ (190) |
| **a [Å]** | 12.6200(18) | 12.6627(18) |
| **b [Å]** | 12.6200(18) | 12.6627(18) |
| **c [Å]** | 18.230(4) | 18.224(4) |
| **α [°]** | 90 | 90 |
| **β [°]** | 90 | 90 |
| **γ [°]** | 120 | 120 |
| **2θ range [°]** | 0.12 to 1.25 (0.90 Å) | 0.10 to 1.26 (0.90 Å) |
| **Index ranges** | −16 ≤ h ≤ 16 −8 ≤ k ≤ 7 −23 ≤ l ≤ 24 | −14 ≤ h ≤ 14 −14 ≤ k ≤ 14 −20 ≤ l ≤ 20 |
| **Independent reflections** | 730 R_int_ = ? R_sigma_ = 0.1883 | 1283 R_int_ = 0.9977 R_sigma_ = 0.1268 |
| **Completeness to  θ = 0.630°** | 99.9 % | 99.9 % |
| **Data / Restraints / Parameters** | 730/3/91 | 1283/55/88 |
| **Goodness-of-fit** | 1.214 | 1.237 |
| **Final R indexes  [I≥2σ(I)]** | *R*_1_ = 0.1833 w*R*_2_ = 0.4139 | *R*_1_ = 0.1398 w*R*_2_ = 0.3418 |
| **Final R indexes  [all data]** | *R*_1_ = 0.1916 w*R*_2_ = 0.4237 | *R*_1_ = 0.1846 w*R*_2_ = 0.3798 |

Table S 9 Input composition and number of crystals identified in the mixture

|  | **Glycine** | **L-ascorbic** | **Zn acetate** | **Saccharin** | **Mg acetate** | **L-glutamic** |
| --- | --- | --- | --- | --- | --- | --- |
| Density (g/cm^3^) | 1.61 | 1.65 | 1.74 | 0.828 | 1.45 | 1.46 |
| Mass (mg) | 50 | 50 | 50 | 50 | 50 | 50 |
| Volume (cm^3^) | 0.0311 | 0.0303 | 0.0289 | 0.0639 | 0.0345 | 0.0342 |
| Expected volume ratio (%) | 14.2 | 13.8 | 13.1 | 27.5 | 15.7 | 15.6 |
| No. crystals | 54 | 87 | 81 | 137 | 65 | 71 |
| Measured volume ratio (%) | 11.0 | 17.5 | 16.3 | 27.7 | 13.2 | 14.3 |
| Measured weight ratio (%) | 13.3 | 20.5 | 19.7 | 16.8 | 14.3 | 15.4 |

Table S 10 Data processing statistics for the mixture

|  | **Glycine** | **L-ascorbic** | **Zn acetate** | **Saccharin** | **Mg acetate** | **L-glutamic** |
| --- | --- | --- | --- | --- | --- | --- |
| Resolution (Å) | 11.96 - 0.95 | 16.71 - 0.95 | 6.97 - 0.95 | 11.34 - 0.95 | 11.68 - 0.95 | 17.21 - 0.95 |
| Redundancy | 9.1 | 9.5 | 27.2 | 36.5 | 3.7 | 6.4 |
| Mean I/σ(I) | 8.6 | 12.8 | 11.1 | 10.9 | 6.8 | 14.5 |
| R_merge_ | 0.515 | 0.484 | 0.604 | 0.569 | 0.47 | 0.47 |
| R_meas_ | 0.541 | 0.51 | 0.611 | 0.576 | 0.528 | 0.506 |
| R_pim_ | 0.149 | 0.151 | 0.08 | 0.086 | 0.223 | 0.178 |
| R_split_ | 0.212 | 0.153 | 0.198 | 0.095 | 0.355 | 0.155 |
| CC½ | 0.916 | 0.971 | 0.96 | 0.995 | 0.749 | 0.978 |

Table S 11 Structure refinement statistics for the mixture

|  | **Glycine** | **L-ascorbic** | **Zn acetate** | **Saccharin** | **Mg acetate** | **L-glutamic** |
| --- | --- | --- | --- | --- | --- | --- |
| Crystal system | monoclinic | monoclinic | monoclinic | monoclinic | monoclinic | orthorhombic |
| Space group | $P2_{1}/n$ (14) | $P2_{1}$ (4) | $C2/c$ (15) | $P2_{1}/c$ (14) | $P2_{1}/c$ (14) | $P2_{1}2_{1}2_{1}$ (19) |
| a [Å] | 5.005(2) | 6.315(2) | 14.394(3) | 9.422(2) | 4.8084(4) | 5.091(2) |
| b [Å] | 11.964(2) | 6.448(2) | 5.330(2) | 6.809(2) | 11.9943(13) | 17.209(3) |
| c [Å] | 5.368(2) | 16.893(3) | 10.962(3) | 11.665(2) | 8.5548(8) | 6.930(2) |
| α [°] | 90.00 | 90.00 | 90 | 90.00 | 90 | 90.00 |
| β [°] | 111.83(3) | 99.01(3) | 99.88(2) | 103.53(3) | 95.355(7) | 90.00 |
| γ [°] | 90.00 | 90.00 | 90 | 90.00 | 90 | 90.00 |
| 2θ range [°] | 0.19 to 1.22 (0.93 Å) | 0.07 to 1.22 (0.93 Å) | 0.16 to 1.17 (0.96 Å) | 0.12 to 1.19 (0.95 Å) | 0.16 to 1.20 (0.94 Å) | 0.13 to 1.19 (0.95 Å) |
| Index ranges | −5 ≤ h ≤ 5 −12 ≤ k ≤ 12 −5 ≤ l ≤ 5 | −6 ≤ h ≤ 6 −6 ≤ k ≤ 6 −17 ≤ l ≤ 17 | −14 ≤ h ≤ 13 −5 ≤ k ≤ 5 −11 ≤ l ≤ 11 | −9 ≤ h ≤ 9 −7 ≤ k ≤ 7 −12 ≤ l ≤ 12 | −4 ≤ h ≤ 4 −12 ≤ k ≤ 12 −8 ≤ l ≤ 8 | −5 ≤ h ≤ 5 −18 ≤ k ≤ 17 −7 ≤ l ≤ 7 |
| Reflections collected | 5767 | 15474 | 11616 | 60945 | 3305 | 9277 |
| Independent reflections | 362 *R*_int_ = 0.5281 *R*_sig_ = 0.1747 | 1635 R_int_ = 0.5784 R_sig_ = 0.2014 | 427 *R*_int_ = 0.6088 *R*_sig_ = 0.2379 | 895 *R*_int_ = 0.5382 *R*_sig_ = 0.0761 | 542 *R*_int_ = 0.6925 *R*_sig_ = 0.3286 | 743 *R*_int_ = 0.7130 *R*_sig_ = 0.1956 |
| Completeness to  θ = 0.609° | 99.5 % | 98.3 % | 88.6 % | 99.7 % | 94.8 % | 100.0 % |
| Data / Restraints / Parameters | 362/0/48 | 1635/1/234 | 427/0/52 | 895/18/110 | 542/0/62 | 743/0/46 |
| Goodness-of-fit | 1.103 | 0.928 | 1.932 | 3.385 | 1.025 | 1.111 |
| Final R indexes  [I≥2σ(I)] | *R*_1_ = 0.1902 w*R*_2_ = 0.4613 | *R*_1_ =0.1732 w*R*_2_ = 0.4105 | *R*_1_ = 0.2095 w*R*_2_ = 0.4476 | *R*_1_ = 0.1616 w*R*_2_ = 0.3458 | *R*_1_ =0.3327 w*R*_2_ = 0.6709 | *R*_1_ = 0.2266 w*R*_2_ = 0.5168 |
| Final R indexes  [all data] | *R*_1_ = 0.2209 w*R*_2_ = 0.4941 | *R*_1_ = 0.2168 w*R*_2_ = 0.4762 | *R*_1_ = 0.3256 w*R*_2_ = 0.5159 | *R*_1_ = 0.2108 w*R*_2_ = 0.3868 | *R*_1_ = 0.4102 w*R*_2_ = 0.7422 | *R*_1_ = 0.2702 w*R*_2_ = 0.5726 |

Table S 12 Summary of the phase analysis results of the crystalline contents in paracetamol tablet and iron supplement capsule using t-SerialED and PXRD. All the ratios displayed in the table are calculated without considering the amorphous contents.

|  | Paracetamol tablet | Iron supplement capsule |
| --- | --- | --- |
| No. of datasets in API unit cells | 163 | 215 (form 1: 207, form 2: 8) |
| No. of datasets in talc unit cells | 1 | 2 |
| Volume ratio of API (ED) | 99.4% | 99.0% (form 1: 95.4%, form 2: 3.6%) |
| Volume ratio of talc (ED) | 0.6% | 1.0% |
| Volume ratio of API (PXRD) | 98.2% | 97.7% (form 1: 97.6%, form 2: 0.1%) |
| Volume ratio of talc (PXRD) | 1.8% | 2.3% |

Table S 13 Data processing statistics for paracetamol. Overall, low and high represent resolution shells.

|  | Overall | Low | High |
| --- | --- | --- | --- |
| High resolution limit | 0.90 | 2.44 | 0.90 |
| Low resolution limit | 11.50 | 11.50 | 0.92 |
| Completeness | 100.0% | 100.0% | 100.0% |
| Redundancy | 22.6 | 47.2 | 13.0 |
| I/sigma | 6.5 | 12.3 | 1.9 |
| *R_merge_* | 0.978 | 1.935 | 1.200 |
| *R_meas_* | 0.996 | 1.957 | 1.255 |
| *R_pim_* | 0.183 | 0.280 | 0.355 |
| *R_split_* | 0.283 | 0.158 | 0.631 |
| *CC_1/2_* | 0.942 | 0.974 | 0.751 |

Table S 14 Data statistics of the refinement results of paracetamol

| Crystal system | monoclinic |
| --- | --- |
| Space group (number) | $P2_{1}/n$ (14) |
| a [Å] | 7.100 |
| b [Å] | 9.210 |
| c [Å] | 11.610 |
| α [°] | 90 |
| β [°] | 97.90 |
| γ [°] | 90 |
| 2θ range [°] | 0.16 to 1.29 (0.88 Å) |
| Index ranges | −7 ≤ h ≤ 7 −10 ≤ k ≤ 10 −12 ≤ l ≤ 12 |
| Reflections collected | 24365 |
| Independent reflections | 1070 R_int_ = 0.8518 R_sigma_ = 0.1728 |
| Completeness to  θ = 0.6443° | 100.0 % |
| Data / Restraints / Parameters | 1070/0/104 |
| Goodness-of-fit on F^2^ | 0.981 |
| Final R indexes  [I≥2σ(I)] | R_1_ = 0.1726 wR_2_ = 0.4187 |
| Final R indexes  [all data] | R_1_ = 0.2165 wR_2_ = 0.4731 |

Table S 15 Data processing statistics for **FGC** sample after filtering datasets below 200 and 100 reflections. Overall, low and high represent resolution shells.

| Threshold: 200 | Overall | Low | High |
| --- | --- | --- | --- |
| High resolution limit | 0.90 | 2.44 | 0.90 |
| Low resolution limit | 13.21 | 13.21 | 0.92 |
| Completeness | 100.0% | 100.0% | 100.0% |
| Redundancy | 10.8 | 30.1 | 5.8 |
| I/sigma | 4.3 | 15.6 | 1.0 |
| *R_merge_* | 0.858 | 0.896 | 1.042 |
| *R_meas_* | 0.892 | 0.908 | 1.182 |
| *R_pim_* | 0.226 | 0.141 | 0.549 |
| *R_split_* | 0.334 | 0.268 | 0.456 |
| *CC_1/2_* | 0.757 | 0.607 | -0.016 |

| Threshold: 100 | Overall | Low | High |
| --- | --- | --- | --- |
| High resolution limit | 0.90 | 2.44 | 0.90 |
| Low resolution limit | 13.21 | 13.21 | 0.92 |
| Completeness | 100.0% | 100.0% | 100.0% |
| Redundancy | 22.1 | 62.2 | 11.0 |
| I/sigma | 4.8 | 18.2 | 1.1 |
| *R_merge_* | 0.775 | 0.855 | 1.359 |
| *R_meas_* | 0.790 | 0.863 | 1.426 |
| *R_pim_* | 0.140 | 0.105 | 0.420 |
| *R_split_* | 0.284 | 0.244 | 0.728 |
| *CC_1/2_* | 0.752 | 0.543 | 0.685 |

Table S 16 Refinement statistics for **FGC** sample after filtering datasets below 200 and 100 reflections.

| **Indexed spots threshold** | **200** | **100** |
| --- | --- | --- |
| **Crystal system** | triclinic | triclinic |
| **Space group (number)** | $P\overline{1}$ (2) | $P\overline{1}$ (2) |
| **a [Å]** | 5.979(2) | 5.979(2) |
| **b [Å]** | 6.793(2) | 6.793(2) |
| **c [Å]** | 13.268(3) | 13.268(3) |
| **α [°]** | 85.49(3) | 85.49(3) |
| **β [°]** | 82.71(3) | 82.71(3) |
| **γ [°]** | 83.09(3) | 83.09(3) |
| **2θ range [°]** | 0.09 to 1.26 (0.90 Å) | 0.09 to 1.26 (0.90 Å) |
| **Reflections collected** | 17290 | 34004 |
| **Independent reflections** | 1537 R_int_ = 0.7388 R_sigma_ = 0.3232 | 1538 R_int_ = 0.7776 R_sigma_ = 0.2630 |
| **Completeness to θ = 0.630°** | 99.1 % | 99.5 % |
| **Data / Restraints / Parameters** | 1537/4/158 | 1538/4/158 |
| **Goodness-of-fit on F^2^** | 1.023 | 1.057 |
| **Final R indexes  [I≥2σ(I)]** | *R*_1_ = 0.2082 w*R*_2_ = 0.4674 | *R*_1_ = 0.1704 w*R*_2_ = 0.4486 |
| **Final R indexes  [all data]** | *R*_1_ = 0.2725 w*R*_2_ = 0.5268 | *R*_1_ = 0.2338 w*R*_2_ = 0.5109 |

Table S 17. Comparison between different methods.


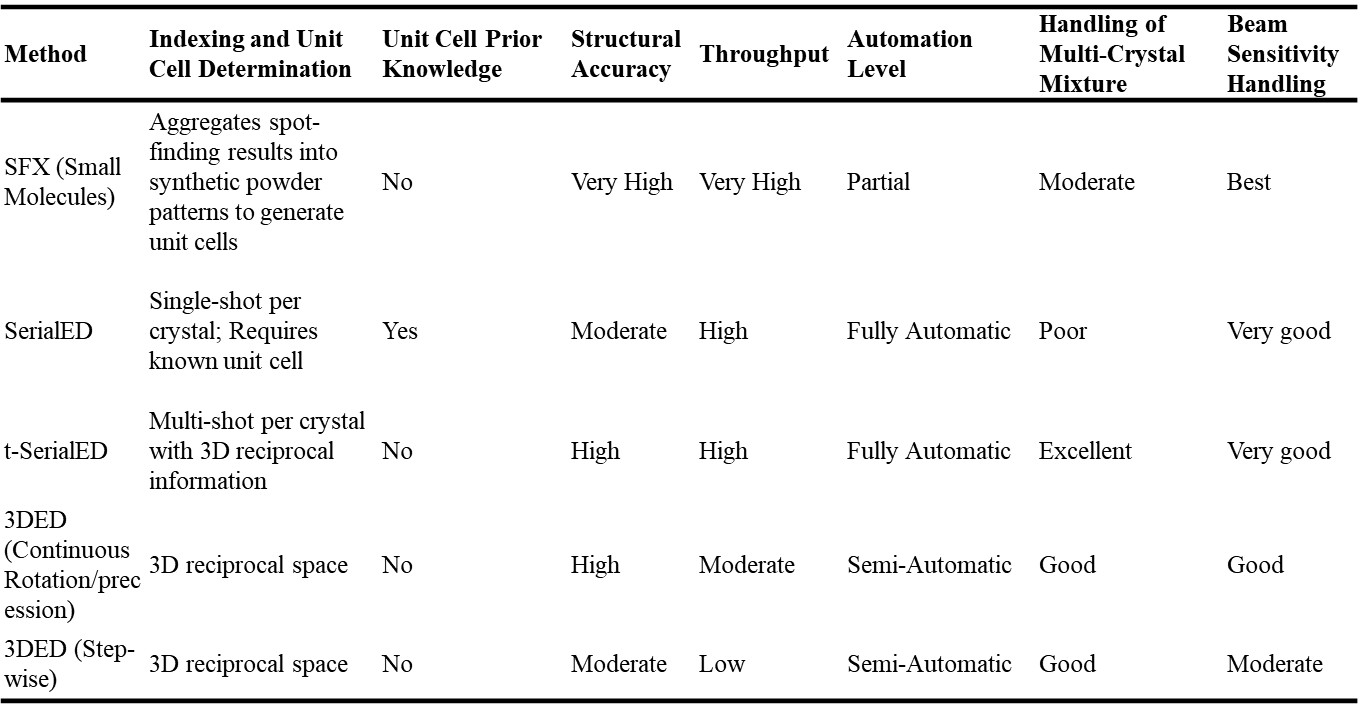


Table S 18. Average deviation from reference atoms for MOF-235 compared with the structure obtained from single crystal X-ray diffraction (10.1021/ic050064g)


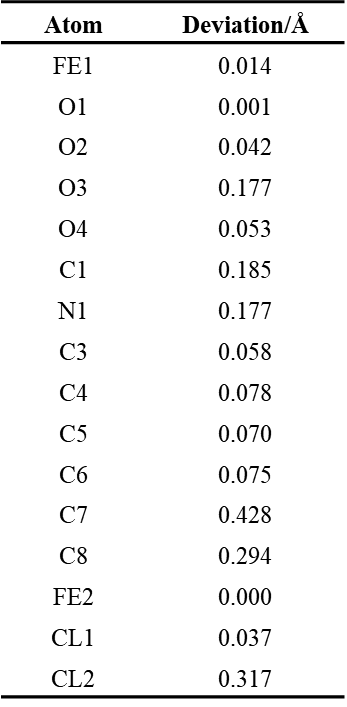


Reference

1. Lee, J. H., Ahn, Y. & Kwak, S.-Y. Facile Sonochemical Synthesis of Flexible Fe-Based Metal–Organic Frameworks and Their Efficient Removal of Organic Contaminants from Aqueous Solutions. *ACS Omega* **7**, 23213–23222 (2022).

2. Bücker, R. *et al.* Serial protein crystallography in an electron microscope. *Nat Commun* **11**, 996 (2020).

3. Bücker, R., Hogan-Lamarre, P. & Miller, R. J. D. Serial Electron Diffraction Data Processing With diffractem and CrystFEL. *Front. Mol. Biosci.* **8**, (2021).

4. Smeets, S., Zou, X. & Wan, W. Serial electron crystallography for structure determination and phase analysis of nanocrystalline materials. *J Appl Cryst* **51**, 1262–1273 (2018).

5. Hogan-Lamarre, P., Luo, Y., Bücker, R., Miller, R. J. D. & Zou, X. STEM SerialED: achieving high-resolution data for ab initio structure determination of beam-sensitive nanocrystalline materials. *IUCrJ* **11**, 62–72 (2024).

6. Wang, B., Zou, X. & Smeets, S. Automated serial rotation electron diffraction combined with cluster analysis: an efficient multi-crystal workflow for structure determination. *IUCrJ* **6**, 854–867 (2019).

7. Luo, Y. *et al.* High-throughput phase elucidation of polycrystalline materials using serial rotation electron diffraction. *Nat. Chem.* **15**, 483–490 (2023).

8. Lightowler, M. *et al.* Phase Identification and Discovery of an Elusive Polymorph of Drug-Polymer Inclusion Complex Using Automated 3D Electron Diffraction. *Angewandte Chemie International Edition* **63**, e202317695 (2024).

9. Takaba, K., Maki-Yonekura, S. & Yonekura, K. Collecting large datasets of rotational electron diffraction with ParallEM and SerialEM. *Journal of Structural Biology* **211**, 107549 (2020).

10. Unge, J., Lin, J., Weaver, S. J., Sae Her, A. & Gonen, T. Compositional Analysis of Complex Mixtures using Automatic MicroED Data Collection. *Advanced Science* **11**, 2400081 (2024).
